# Combinatorial expression of *Lef1, Lhx2, Lhx5, Lhx9, Lmo3, Lmo4*, and *Prox1* helps to identify comparable subdivisions in the developing hippocampal formation of mouse and chicken

**DOI:** 10.3389/fnana.2014.00059

**Published:** 2014-07-04

**Authors:** Antonio Abellán, Ester Desfilis, Loreta Medina

**Affiliations:** Laboratory of Brain Development and Evolution, Department of Experimental Medicine, Institute of Biomedical Research of Lleida, University of LleidaLleida, Spain

**Keywords:** medial pallium, hippocampus, dentate gyrus, Ammon's horn fields, entorhinal cortex, dorsolateral caudal pallium, evolution

## Abstract

We carried out a study of the expression patterns of seven developmental regulatory genes (*Lef1, Lhx2, Lhx9, Lhx5, Lmo3, Lmo4*, and *Prox1*), in combination with topological position, to identify the medial pallial derivatives, define its major subdivisions, and compare them between mouse and chicken. In both species, the medial pallium is defined as a pallial sector adjacent to the cortical hem and roof plate/choroid tela, showing moderate to strong ventricular zone expression of *Lef1, Lhx2*, and *Lhx9*, but not *Lhx5*. Based on this, the hippocampal formation (*indusium griseum*, dentate gyrus, Ammon's horn fields, and subiculum), the medial entorhinal cortex, and part of the amygdalo-hippocampal transition area of mouse appeared to derive from the medial pallium. In the chicken, based on the same position and gene expression profile, we propose that the hippocampus (including the V-shaped area), the parahippocampal area (including its caudolateral part), the entorhinal cortex, and the amygdalo-hippocampal transition area are medial pallial derivatives. Moreover, the combinatorial expression of *Lef1, Prox1, Lmo4*, and *Lmo3* allowed the identification of dentate gyrus/CA3-like, CA1/subicular-like, and medial entorhinal-like comparable sectors in mouse and chicken, and point to the existence of mostly conserved molecular networks involved in hippocampal complex development. Notably, while the mouse medial entorhinal cortex derives from the medial pallium (similarly to the hippocampal formation, both being involved in spatial navigation and spatial memory), the lateral entorhinal cortex (involved in processing non-spatial, contextual information) appears to derive from a distinct dorsolateral caudal pallial sector.

## Introduction

The hippocampal formation is a cortical structure of the telencephalic hemispheres that is essential for spatial navigation and memory formation (Bird and Burgess, 2008). Interest in this region mainly comes from data in mammals showing that: (1) Damage to the hippocampal formation (as it occurs in Alzheimer's disease) produces a decline or have devastating effects in spatial navigation and memory (Bird and Burgess, 2008; Lithfous et al., 2013). (2) The hippocampal formation (in particular, the dentate gyrus) is one of the few brain regions showing adult neurogenesis (Alvarez-Buylla and Lim, 2004; Gould, 2007), which has been related to network plasticity, learning and memory formation, and the ability to adapt to novelty and complexity (Gould et al., 1999; Leuner et al., 2006; Kempermann, 2008; Varela-Nallar and Inestrosa, 2013; Vivar and van Praag, 2013). (3) Dysfunction of the hippocampal formation and dysregulation of adult hippocampal neurogenesis are associated to several mental disorders and neurological diseases (Eisch et al., 2008; DeCarolis and Eisch, 2010; Jun et al., 2012; Mendez-David et al., 2013).

In mammals, the hippocampal formation (HF) comprises three cytoarchitectonically distinct subdivisions, which from lateral to medial are: the subiculum, the hippocampus proper (Ammon's horn fields or cornu ammonis, subdivided in CA1, CA2, and CA3 fields) and the dentate gyrus (reviewed in Witter and Amaral, 2004; Witter, 2012). It also includes a rostral continuation called *indusium griseum* (Künzle, 2004). Within the HF, each subdivision is unique regarding its histological, neurochemical and connectivity patterns (Witter and Amaral, 2004; Witter, 2012). The projections of the dentate gyrus and CA fields are mostly intrinsic and associational (within the ipsilateral and contralateral HF), while the subiculum (with a small contribution of CA1) is the major output hippocampal center, with projections to several cortical and subcortical regions, hypothalamus, and midline thalamus (Witter and Amaral, 2004). The HF receives input from the medial septum, nucleus of the diagonal band, pallial amygdala, hypothalamus, midline thalamus, and several brainstem monoaminergic cell groups. Most importantly, the HF is reciprocally connected with the entorhinal cortex, which is part of the parahippocampal region (Witter and Amaral, 2004). Due to the strong functional relationship between the HF and the parahippocampal region, both regions are often included as parts of the hippocampal functional complex, although they differ in other respects, such as position, cytoarchitecture, neurochemistry, and connections (Witter and Amaral, 2004; Witter, 2012). The entorhinal cortex is an essential actor for hippocampal functions, and is also extensively and reciprocally connected with the neocortex, pallial and subpallial-extended amygdala, and septum/diagonal band nuclei (Witter and Amaral, 2004).

Classical studies described in detail the development of rat HF subdivisions from distinct progenitor sectors of the medial pallium (Altman and Bayer, 1990a,b,c). More recently, the molecular control of HF development has started to be elucidated. Wnt and BMP signals from the cortical hem, roof plate and/or meninges are essential for HF development (Galceran et al., 2000; Lee et al., 2000; Machon et al., 2007; Choe et al., 2013), and these induce the expression in the medial pallium of several transcription factors important for different aspects of HF development, such as Lhx2 (Porter et al., 1997; Bulchand et al., 2001; Monuki et al., 2001; Vyas et al., 2003), Lef1 (lymphoid enhancer factor 1) and other TCF transcription factors (Galceran et al., 2000; Choe et al., 2013). In particular, Lef1 is crucial for the production of dentate gyrus granule cells, and Lef1 together with other TCFs are necessary for the development of the whole HF, which is not formed following subrogation of their function (Galceran et al., 2000). Wnt and Lef1 induce the expression of another transcription factor specifically in the dentate gyrus, Prox1 (prospero-related homeobox 1 gene), which is involved in the differentiation of granule cells (Zhou et al., 2004; Lavado et al., 2010; Iwano et al., 2012). Interestingly, some of the regulatory genes involved in the development of the HF, such as those encoding some molecules of the Wnt/β catenin pathway, some TCFs (mostly TCF4), and Prox1, continue to be expressed and are functional in the adult dentate gyrus (Shimogori et al., 2004; Karalay et al., 2011), and at least Wnt signaling and Prox1 play important roles in distinct aspects of adult neurogenesis, such as cell proliferation or the differentiation of new granule cells (Karalay et al., 2011; Iwano et al., 2012; Varela-Nallar and Inestrosa, 2013). In addition to its role in granule cell specification, differentiation, and survival (reviewed by Karalay and Jessberger, 2011), recent data in mouse showed that, from late embryonic stages, Prox1 is also expressed in subsets of neocortical and hippocampal interneurons, which derive from the caudolateral ganglionic eminence and the preoptic area of the subpallium (Rubin and Kessaris, 2013). In spite of the abundance of data on HF development, very little is known on the genes that regulate the development of the entorhinal cortex in mammals.

A hippocampal formation, involved in spatial navigation and memory formation, has been identified in a topologically comparable pallial position in non-mammalian amniotes (sauropsids, i.e., birds and reptiles) and in several anamniotes (reviewed by Rodríguez et al., 2002; Reiner et al., 2004). Comparative studies using an evolutionary developmental biology (evodevo) approach are turning extremely useful for understanding not only the origin but also many aspects of both the anatomical and functional organization of brain regions (Puelles and Medina, 2002; Medina and Abellán, 2009; Medina et al., 2011; Abellán et al., 2013). However, very little is known on the regulatory genes involved in the development of the HF and entorhinal cortex in non-mammals. Herein, we carried out a comparative study of the combinatorial mRNA expression patterns of *Lef1*, several *LIM*-homeobox (*Lhx2, Lhx5*, and *Lhx9*) and *LIM*-only (*Lmo3* and *Lmo4*) genes, and *Prox1* in the developing medial pallium of mouse and chicken. Although the expression of all of these genes was previously studied in the developing dorsomedial pallium of mouse (Galceran et al., 2000; Bulchand et al., 2001, 2003; Zhou et al., 2004; Abellán et al., 2009, 2010; Lavado et al., 2010), herein we analyzed in detail their combinatorial expression patterns in order to: (1) distinguish molecularly the whole ventricular sector of the medial pallium and the different structures it produces in mouse; (2) discern whether the entorhinal cortex develops from the medial pallium or from another embryonic pallial sector; and (3) compare these patterns in mouse with those of the orthologous genes in chicken, as a contribution to understand hippocampal evolution. Our data allowed the identification of dentate gyrus/CA3-like, CA1/subicular-like, and entorhinal-like comparable sectors in mouse and chicken, and point to the existence of mostly conserved molecular networks involved in hippocampal complex development.

## Materials and methods

Mouse embryos (Swiss) from embryonic day 11.5 (E11.5) until birth and chicken embryos from embryonic day 6 (E6, HH29) until 2 days after hatching (P2) were used in the present study. All animals were treated according to the regulations and laws of the European Union (86/609/EEC) and the Spanish Government (Royal Decree 1021/2005) for care and handling of animals in research. The protocols used were approved by the Committee for handling and care of research animals of the University of Lleida. The mouse embryos were obtained from pregnant females, and were processed and fixed as previously described (García-López et al., 2008). The chicken embryos were obtained from fertilized eggs bought in a farm, which were incubated in a forced-draft incubator until the desired embryonic stage. The chicken embryos were staged according to Hamburger and Hamilton (1951). Upon extraction, the brains of earlier embryos (E11.5–E15.5 in mouse; 6–11 days incubation in chicken: E6–E11 or HH29-HH37) were dissected and fixed by immersion in 4% paraformaldehyde diluted in 0.1 M phosphate-buffered saline (pH 7.5; PBS) at 4°C during 24 h. Older embryos (E16.5–E18.5 in mouse; from E12 or HH38 to pre-hatching in chicken) and P0–P2 animals were first deeply anesthetized with sodium pentobarbital (Dolethal, 15 mg/kg), and perfused transcardially with NaCl saline solution (0.9% for mouse; 0.75% for chicken), followed by phosphate-buffered 4% paraformaldehyde (pH 7.5). The brains were then dissected and postfixed overnight at 4°C. After fixation, the brains were embedded in 4% agarose in PBS, sectioned at 80–120 μm for *in situ* hybridization in the transversal or horizontal planes using a vibratome (Leica VT1000S), and were subsequently processed as floating sections.

### *In situ* hybridization

Brain sections were processed for *in situ* hybridization following a variation of the standard procedure using digoxigenin-labeled riboprobes (Medina et al., 2004; García-López et al., 2008; Abellán and Medina, 2009). The riboprobes were synthesized from cDNAs of different mouse or chicken genes.

The cDNAs from mouse genes were obtained from other laboratories:

*Lef1* (Galceran et al., 2000; bp 1–729; Genbank accession no: NM_010703);
*Lhx2* (plasmid obtained from S. Rétaux's lab; Rétaux et al., 1999; bp 1–1300; Genbank accession no: NM_010710.3);
*Lhx5* (plasmid obtained from H. Westphal's lab; Zhao et al., 1999; bp 1–2226; Genbank accession no: U61155.1);
*Lhx9* (plasmid obtained from S. Rétaux's lab; Rétaux et al., 1999; bp 1–1016 [full lenght]; Genbank accession no: AF134761);
*Lmo3* (plasmid obtained from J.L.R. Rubenstein's lab; Bulchand et al., 2001; bp 1–2101 [full lenght]; Genbank accession no: NM_207222);
*Lmo4* (plasmid obtained from J.L.R Rubenstein's lab; Bulchand et al., 2001; bp 1–498 [full lenght]; Genbank accession no: AF074600).

The cDNAs from chicken genes were purchased [cDNA ESTs purchased from ARK-genomics (Roslin Institute; Midlothian, UK) or Geneservice Limited (Cambridge, UK)], or obtained from other laboratories, as indicated below. The purchased clones were obtained from the BBSRC ChickEST Database (Boardman et al., 2002), and have a corresponding Genbank accesssion number:

*cLef1* (bp 1–901; GenBank accession no: CR391621.1; purchased; BBSRC ChickEST Database: clone ChEST891i13);
*cLhx2* (Abellán et al., 2009; bp 208–939; Genbank accession no: NM_204889);
*cLhx5* (Abellán et al., 2010; bp 49–1042; Genbank accession no: XM_001234552);
*cLhx9* (Abellán et al., 2009; bp 596–1502; Genbank accession no: NM_205426);
*cLmo3* (Abellán and Medina, 2009; bp 1–666; Genbank accession no: CR406209; purchased; BBSRC ChickEST Database: clone ChEST853b21);
*cLmo4* (Abellán and Medina, 2009; purchased; bp 307–1078; Genbank accession no: AF532926; purchased; BBSRC ChickEST Database: clone ChEST54p6);
*cProx1* (bp 1–841; GenBank accession no: BU214594; purchased; BBSRC ChickEST Database: clone ChEST49e24).
*cWnt8b* (641 bp; Hollyday et al., 1995; Garda et al., 2002; Genbank accession no: NC_006093.3).

We used PCR to obtain the DNA template employed for synthesizing the riboprobe. We synthesized the antisense digoxigenin-labeled riboprobes using Roche Diagnostics's (Mannheim, Germany) protocols for the genes mentioned above. Before hybridization, the sections were abundantly washed in PBS containing 0.1% Tween-20 (PBT 1X), prehybridized in hybridization buffer (HB) for 2 h at 58°C, and then hybridized in HB containing the riboprobe overnight at 58°C (0.5–1 μg/ml, depending on the probe and embryo size). The hybridization buffer contained 50% of deionized formamide, 1.3X standard saline citrate (SSC; pH 5), 5 mM ethylene-diamine-tetraacetic acid (EDTA; pH 8.0; Sigma-Aldrich, Steinheim, Germany), 1 mg/ml of yeast tRNA (Sigma-Aldrich), 0.2% Tween-20, 100 μg/ml of heparin (Sigma-Aldrich), completed with water (free of RNAase and DNAase; Sigma-Aldrich). Following hybridization, the sections were washed with a mix 1:1 of MABT 1X (1.2% maleic acid, 0.8% NaOH, 0.84% NaCl, and 0.1% Tween-20) and HB at 58°C during 20 min and washed abundantly at room temperature with MABT 1X (about 2 h). Following this, the sections were blocked with a solution containing blocking reagent (Roche), MABT 1X and sheep serum (Sigma) for 4 h at room temperature, then incubated in an antibody against digoxigenin (alkaline-phosphatase coupled anti-digoxigenin; diluted 1:3500; Roche Diagnostics) overnight at 4°C, later washed with MABT 1X and finally revealed with BM purple (Roche Diagnostics). Sections were then mounted on glycerol gelatine (Sigma).

### Immunohistochemistry

Some series of chicken embryonic brain sections (E8–E12) were processed for immunohistochemistry, following a procedure previously described (Abellán and Medina, 2009).

In order to detect radial glial fibers in chicken, we used a monoclonal antibody against chicken vimentin (H5 from Developmental Hybridoma Bank, Iowa, USA; Herman et al., 1993). The specificity of this antibody has beed shown by the manufacturer using Western blot (labeling a band of roughly 52 kDa, corresponding to the protein vimentin).

The immunohistochemical procedure was as follows. After washing in PBS, the sections were incubated in the primary antibody, diluted 1:50 in PBS containing 0.3% Triton X-100, for 2 days at 4°C, under constant and gentle agitation. Then, the sections were washed and incubated in a secondary antiserum for 1 h at room temperature (biotinylated goat anti-mouse IgG; diluted 1:200; Vector, Burlingame, CA, USA). Following this, the sections were washed and incubated in the avidin-biotin complex (ABC kit; Vector; 0.003% dilution) for 1 h at room temperature. Finally, the immunolabeling was revealed by 0.05% diaminobenzidine (DAB; Sigma-Aldrich, Steinheim, Germany) in 0.05 M Tris buffer (pH 7.6), containing 0.03% H_2_O_2_.

### Digital images and figures

Digital photographs were taken on a Leica microscope (DMR HC) equipped with a Zeiss Axiovision digital camera. Digital images were adjusted for brightness/contrast using Adobe PhotoShop and figures were mounted and labeled using Macromedia FreeHand 10.

### Nomenclature

Finally, the nomenclature used in the present study for the chicken telencephalon generally followed that proposed by Reiner et al. (2004), except for developmental units, hippocampal subdivisons, and the entorhinal cortex, for which it followed Redies et al. (2001), Puelles et al. (2007), Abellán et al. (2009). For the mouse embryonic brain we primarily followed Jacobowitz and Abbott (1997), and for the mature mouse hippocampal complex, we followed Paxinos and Franklin (2004) and Witter (2012).

## Results

**Figure 1:**
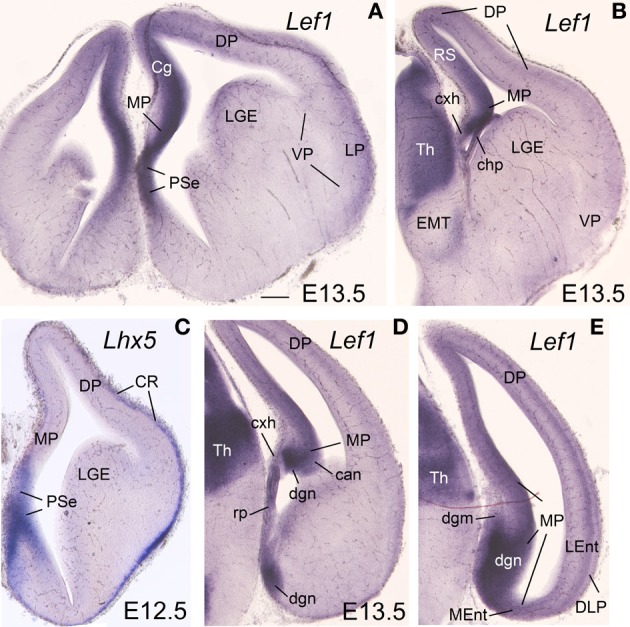
**Expression of *Lef1* in mouse embryonic telencephalon at E13.5.** Digital images of coronal sections of mouse embryonic telencephalon (E13.5), from rostral **(A)** to caudal **(E)** levels, hybridized for *Lef1*. Note the strong expression in the ventricular zone of the medial pallium and pallial septum. For abbreviations see list. Scale bar: **(A)** = 200 μm (applies to all).

**Figure 2:**
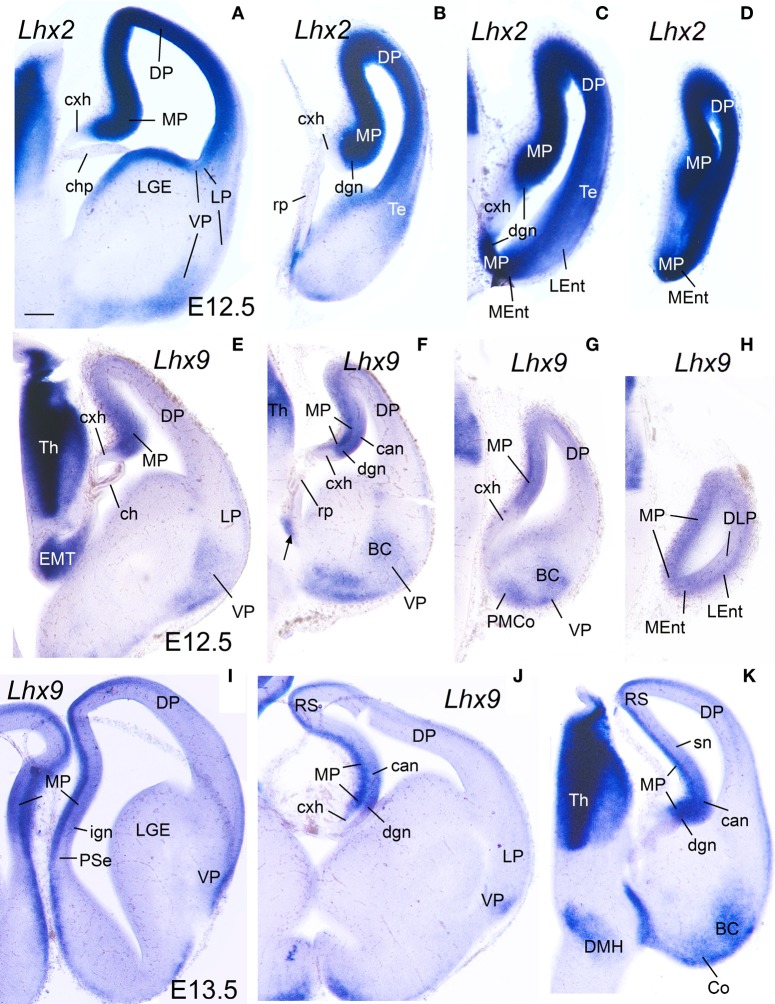
**Expression of *Lhx2* and *Lhx9* in mouse embryonic telencephalon at early stages.** Digital images of coronal sections of mouse embryonic telencephalon (**A–H**: E12.5; **I–K**: E13.5), from intermediate (left panels) to caudal (right panels) levels, hybridized for *Lhx2* **(A–D)** or *Lhx9* **(I–K)**. Note the strong expression in the ventricular zone of the medial pallium. As noted previously, *Lhx9* is also distinctly expressed in ventral pallial (VP) derivatives, such as part of the basal amygdalar complex (BC) and cortical amygdalar areas (Co, PMCo). Although weak transient expression is also present in part of the dorsal pallium (DP; Rétaux et al., 1999), this pallial sector is clearly distinguished from MP and VP based on its distinct position and combinatorial genetic profile (Puelles et al., 2000; Abellán et al., 2009). For abbreviations see list. Scale bar: **(A)** = 200 μm (applies to all).

**Figure 3:**
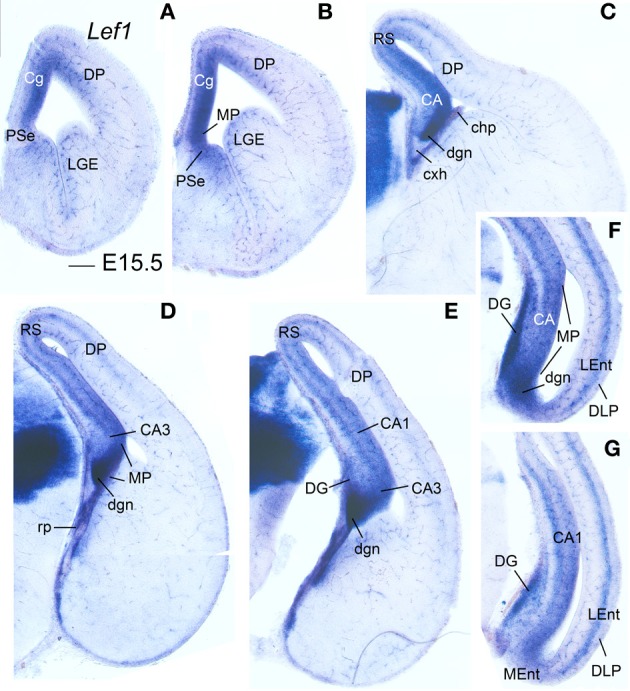
**Expression of *Lef1* in mouse embryonic telencephalon at E15.5.** Digital images of coronal sections of mouse embryonic telencephalon (E15.5), from rostral **(A)** to caudal **(G)** levels, hybridized for *Lef1*. The medial pallial (MP) vz and derivatives show expression of *Lef1*. Note the lack of *Lef1* expression in the vz of the dorsolateral caudal pallium (DLP), giving rise to LEnt. For abbreviations see list. Scale bar: **(A)** = 200 μm (applies to all).

**Figure 4:**
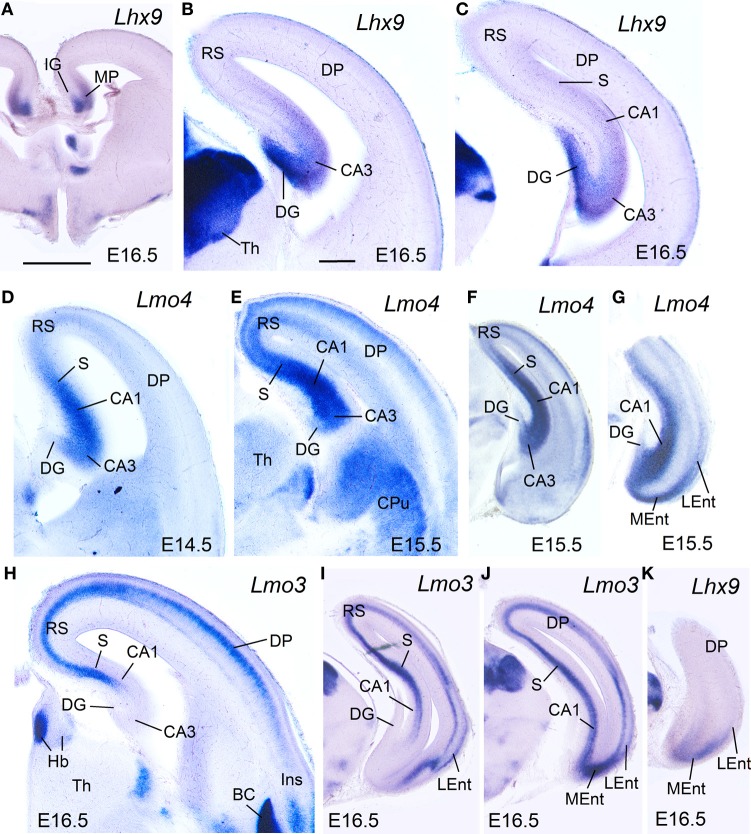
**Expression of *Lhx9, Lmo3*, and *Lmo4* in mouse embryonic telencephalon at intermediate stages.** Digital images of coronal sections of mouse embryonic telencephalon (E15.5 or E16.5), at intermediate **(A–E,H)** or caudal **(F,G,I–K)** levels, hybridized for *Lhx9, Lmo3*, or *Lmo4*. For abbreviations see list. Scale bar: **(A)** = 1 mm (applies to **A,F,G,I–K**); **(B)** = 300 μm (applies to **B–E,H**).

**Figure 5:**
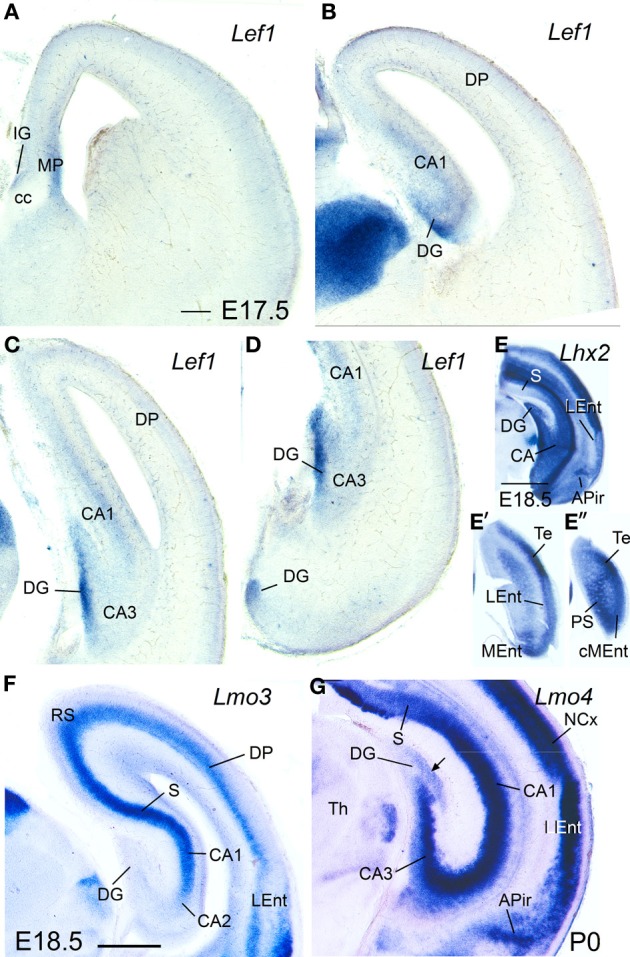
**Expression of *Lef1, Lhx2, Lmo3*, and *Lmo4* in mouse embryonic telencephalon at prenatal or neonatal ages.** Digital images of coronal sections of prenatal or neonatal mouse telencephalon (E17.5, E18.5, or P0), at intermediate **(A,B,F)** or caudal **(C–E″,G)** levels, hybridized for *Lef1, Lhx2, Lmo3*, or *Lmo4*. For abbreviations see list. Scale bar: **(A)** = 200 μm (applies to **A–D,F,G**). **(E)** = 1 mm (applies to **E–E″**).

**Figure 6:**
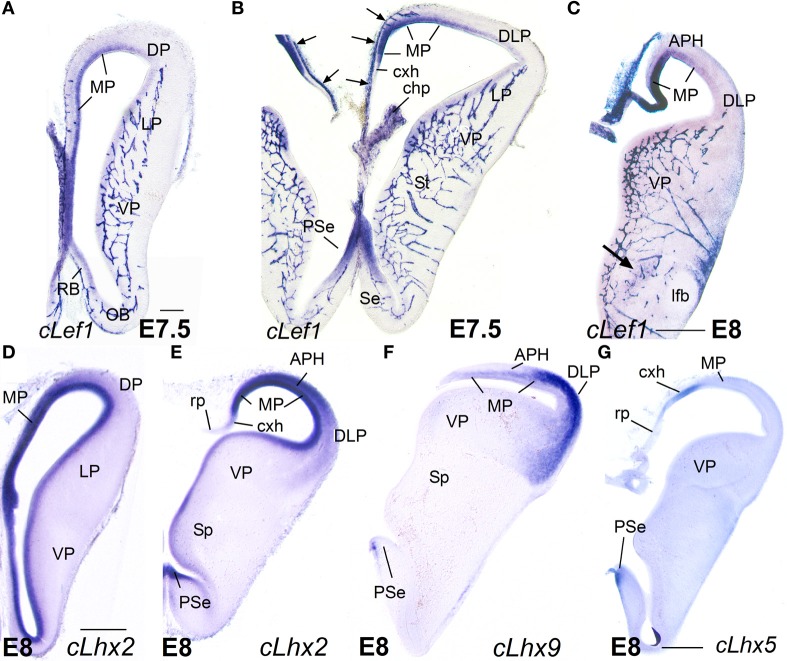
**Expression of *cLef1, cLhx2, cLhx9*, and *cLhx5* in chicken embryonic telencephalon at early stages.** Digital images of coronal sections of chicken embryonic telencephalon (E7.5 or E8), at rostral **(A,D)** or intermediate **(B,C,E–G)** levels, hybridized for *cLef1, cLhx2, cLhx9*, or *cLhx5*. Note the moderate to strong expression of *cLef1, cLhx2*, and *cLhx9* in the ventricular zone of the medial pallium (MP) and pallial septum (PSe). The pallial septum also expresses *cLhx5*. From E8, the dorsolateral pallium (DLP) can be distinguished from MP because it does not express *cLef1*, but shows moderate to strong expression of *cLhx9* in the mantle. Note the expression of *cLef1* in the meninges (*pia mater*; arrows in **B**), in forebrain blood vessels, and in some cell aggregates around the lateral forebrain bundle (arrow in **C**). For abbreviations see list. Scale bar: **(A)** = 200 μm (applies to **A,B**); **(C)** = 400 μm; **(D)** = 500 μm (applies to **D–F**); **(G)** = 500 μm.

**Figure 7:**
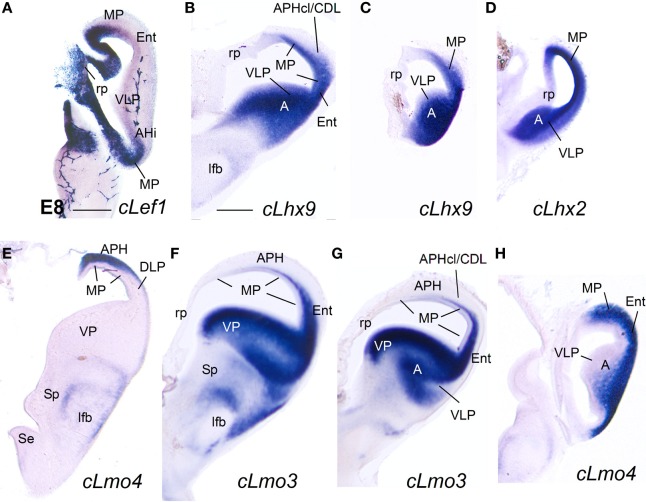
**Expression of *cLef1, cLhx2, cLhx9, cLmo3*, and *cLmo4* in the chicken embryonic telencephalon at early stages.** Digital images of coronal sections of chicken embryonic telencephalon (E8), at intermediate **(E,F)** or caudal **(A–D,G,H)** levels, hybridized for *cLef1, cLhx2, cLhx9, cLmo3*, or *cLmo4*. The medial pallium is characterized by strong expression of *cLef1, cLhx2*, and *cLhx9* in the ventricular zone, and strong expression of *cLhx2* and *cLmo4* in the mantle. For abbreviations see list. Scale bar: **(A)** = 400 μm; **(B)** = 500 μm (applies to **B–H**).

**Figure 8:**
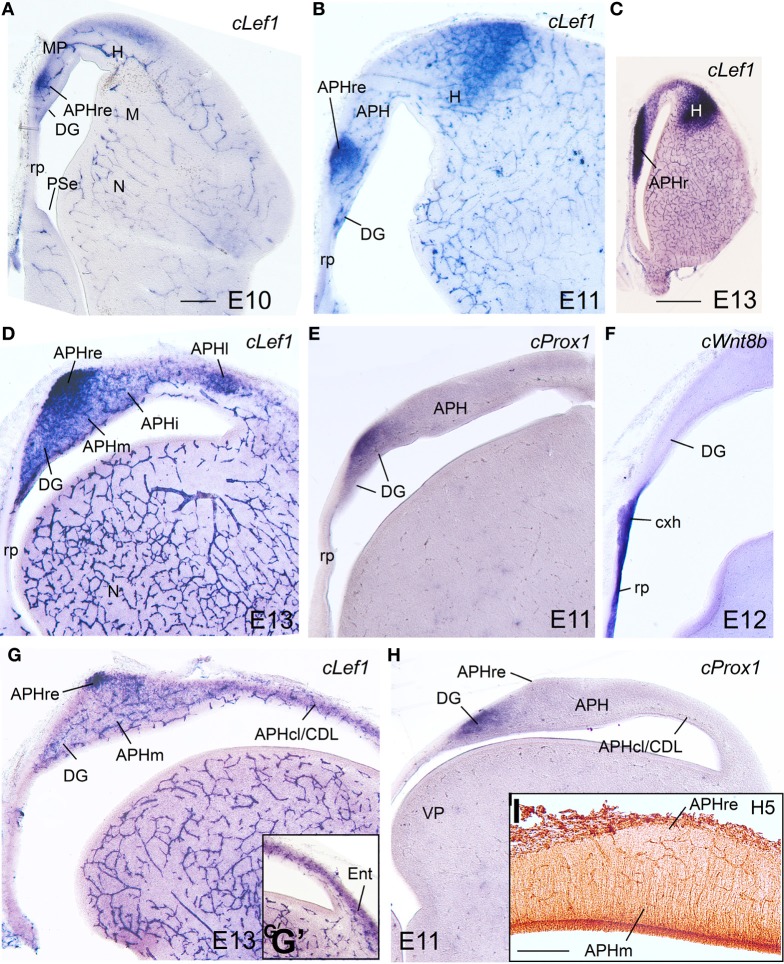
**Expression of several genes and radial glial fibers in the chicken embryonic medial pallium at intermediate stages. (A–H)** Digital images of coronal sections of chicken embryonic telencephalon (E10–E13), at rostral **(A–C)**, intermediate **(D–F)**, or caudal **(G–I)** levels, hybridized for *cLef1, cProx1*, or *cWnt8b* (the latter is used to distinguish the roof plate and cortical hem) Note the strong expression of *cLef1* in the rostral APH, which extends caudally to a small area that occupies a superficial position above APHm. *cProx1* allows distinction of the dentate gyrus (DG). I: Detail of radial glial fibers in the APH (immunohistochemical staining using H5 antibody). Note that the caudal extension of APHr (ectopic APHr or APHre in **G–I**) is avoided by fibers. For abbreviations see list. Scale bar: **(A)** = 200 μm (applies to **A,B,D–H**); **(C)** = 1 mm, **(I)** = 200 μm.

**Figure 9:**
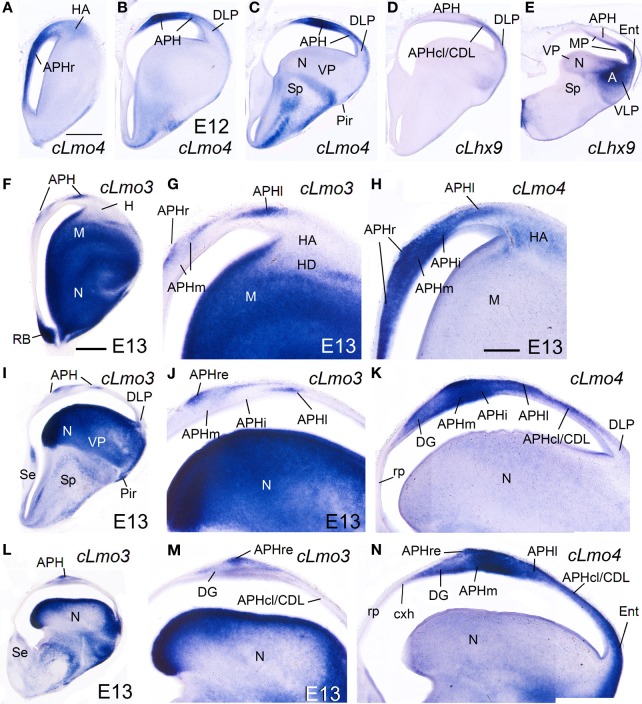
**Expression of several genes in the chicken embryonic telencephalon at intermediate stages.** Digital images of coronal sections of chicken embryonic telencephalon (E12, E13), at rostral **(A,F–H)**, intermediate **(B–D, I–K)** or caudal **(E, L–N)** levels, hybridized for *cLhx9, cLmo3*, or *cLmo4*. Note the strong expression of *cLmo4* in medial pallial derivatives, which is remarkable in APHm and APHi. *cLhx9* is also expressed in the vz of the medial pallium, and at caudal levels the expression becomes stronger and is additionally present in the mantle. Moreover, *cLhx9* is expressed in derivatives of the ventral pallium (VP; in particular, the caudal nidopallium, **N**), and in both vz and derivatives of the ventrolateral caudal pallium (VLP, which gives rise to the arcopallium). For abbreviations see list. Scale bar: **(A)** = 1 mm (applies to **A–E**); **(F)** = 1 mm (applies to **F,I,L**); **(H)** = 500 μm (applies to **G,H,J,K,M,N**).

**Figure 10:**
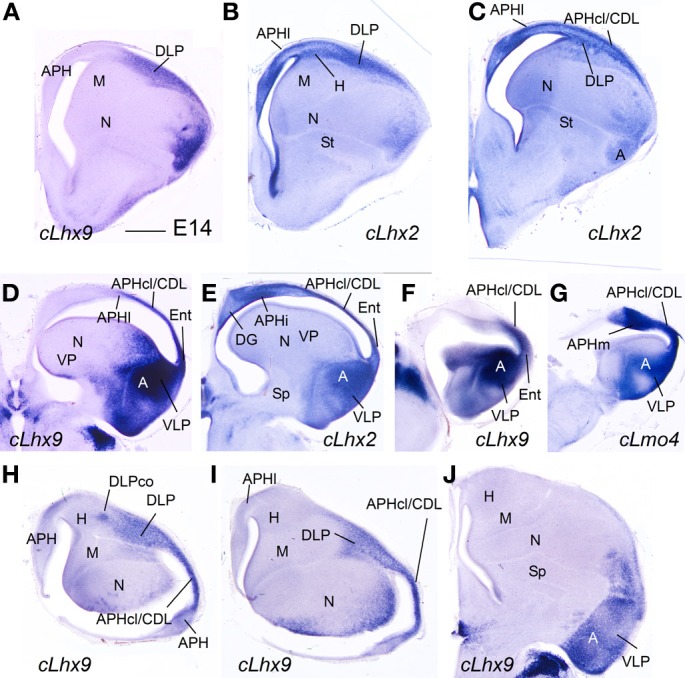
**Expression of several genes in the chicken embryonic telencephalon at intermediate stages. (A–G)** Digital images of coronal sections of chicken embryonic telencephalon (E14), at intermediate **(A–C)** or caudal **(D–G)** levels, hybridized for *cLhx2, cLhx9*, or *cLmo4*. **(H–J)** Digital images of horizontal sections of chicken embryonic telencephalon (E14), from top **(H)** to bottom **(J)**, hybridized for *cLhx9*. Note the distinct genetic profile of DLP and VLP. For abbreviations see list. Scale bar: **(A)** = 1 mm (applies to all).

**Figure 11:**
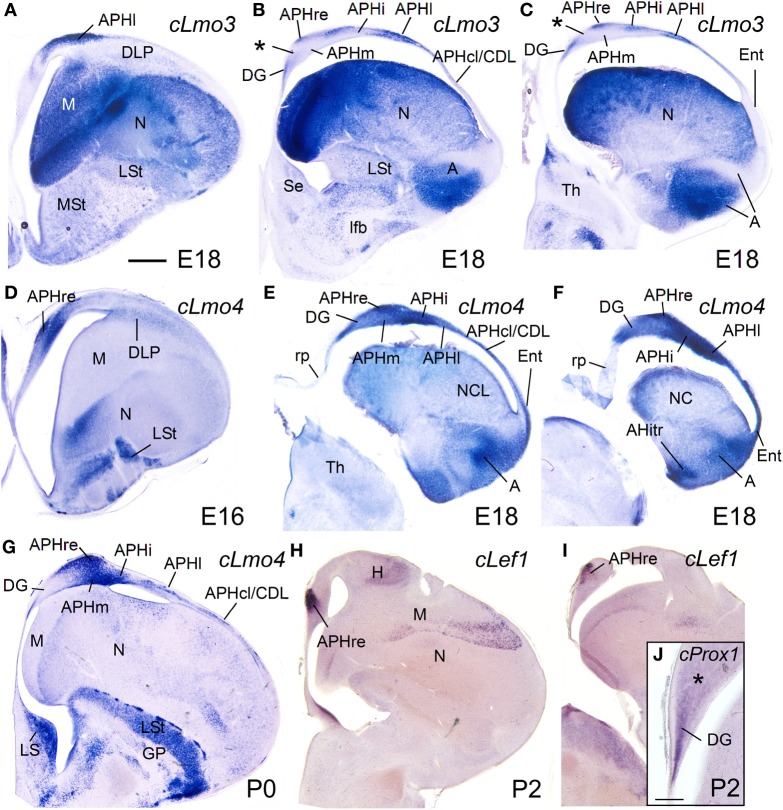
**Expression of several genes in the chicken embryonic telencephalon at prehatching and early posthatching stages.** Digital images of coronal sections of chicken telencephalon (E16, E18, P0, or P2), at intermediate **(A,D,G,H)** or caudal **(B,C,E,F,I,J)** levels, hybridized for *cLmo3, cLmo4, cLef1*, or *cProx1*. Note the expression of cProx1 in the dentate gyrus, which includes a large part of V-shaped area, but not its dorsal part (asterisk in **J**). This dorsal part of V-shaped area (hippocampal sector 2) is also free of *cLmo3* (asterisk in **B,C**) and may be comparable to CA3 of mammals. For abbreviations see list. Scale bar: **(A)** = 1 mm (applies to **A–I**); **(J)** = 200 μm.

Herein we present data on the expression of *Lef1, Lhx2, Lhx9, Lmo3*, and *Lmo4* in the mouse embryonic medial pallium (summarized in Table 1, and shown in Figures 1–**5**), and data on the expression of *cLef1, cLhx2, cLhx9, cProx1, cLmo3*, and *cLmo4* in the chicken embryonic medial pallium (summarized in Table 2, and shown in **Figures 6**–**11**). The figures are organized according to both the species and the age, showing first those for the mouse and then those for the chicken, and within each species showing first those of early embryonic stages, followed by intermediate stages and finally those for late stages. For comparative purposes we also included published data on *Prox1* in mouse in Table 1 (Zhou et al., 2004; Lavado et al., 2010, and the Allen Developing Mouse Brain Atlas). To assist in the distinction of the medial pallial ventricular sector from other pallial sectors, we also analyzed *Lhx5* at early developmental stages in mouse and chicken (Tables 1, 2). In addition to its expression in the pallium, and as noted previously (Oosterwegel et al., 1993; Galceran et al., 2000; Gupta et al., 2012; Choe et al., 2013), *Lef1* was also expressed in other forebrain regions such as the thalamus (Figures 1D,E), as well as in the mesoderm and *pia mater* (neural crest-derived part of the meninges) covering the forebrain during development (arrows in **Figure 6B**), in the developing choroid plexus, and in forebrain blood vessels (**Figures 3**, **6A–C**).

**Table 1:**
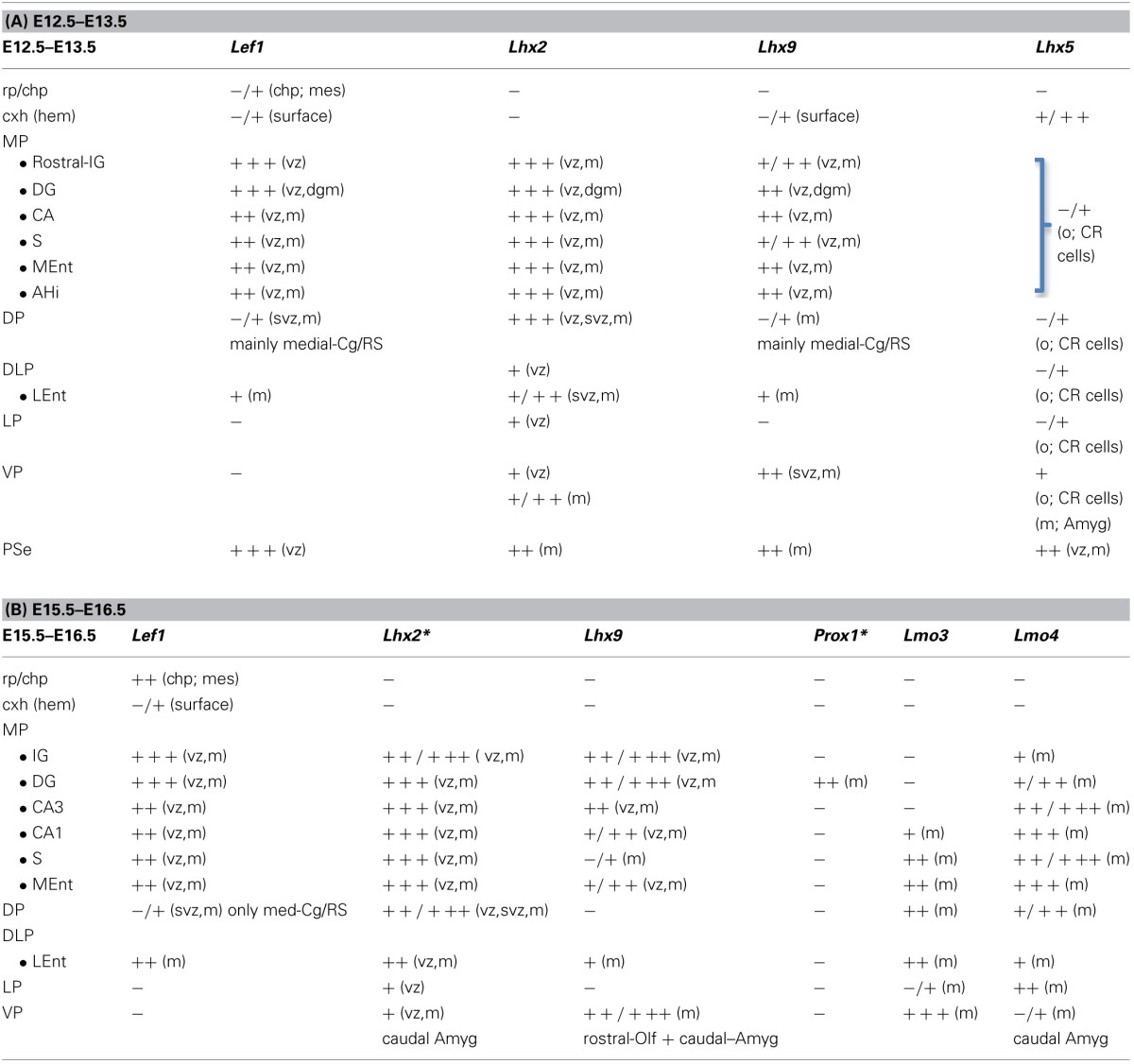
**Combinatorial expression of *Lef1* and other regulatory genes in the pallial progenitor zones and hippocampal complex primordia of developing mice**.

−, no expression; +, weak expression; ++, moderate expression; +++, strong expression.

chp, choroid plexus; dgm, dentate gyrus migratory stream; m, mantle; o, outer or marginal zone; rp, roof plate; svz, subventricular zone; vz, ventricular zone. For other abbreviations see list.

^*^Based on published data (Prox1: Zhou et al., 2004; Lavado et al., 2010; Lhx2: Allen Brain Atlas web site).

**Table 2:**
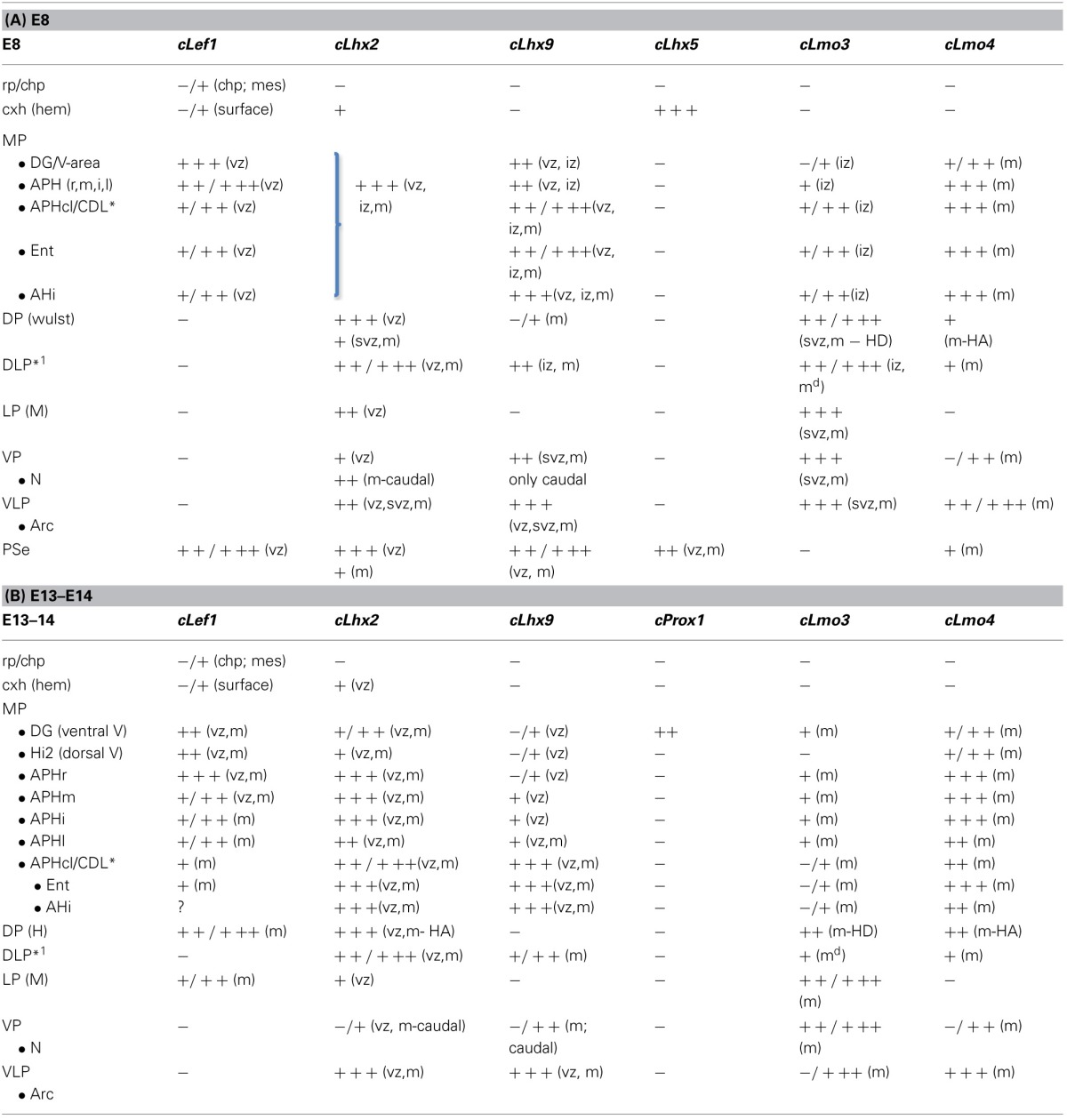
**Combinatorial expression of *Lef1* and other regulatory genes in the pallial progenitor zones and hippocampal complex primordia of developing chicken**.

*−, no expression; +, weak expression; ++, moderate expression; +++, strong expression*.

*chp, choroid plexus; iz, intermediate zone; m, mantle; m^d^, deep part of mantle; mes, mesoderm; rp, roof plate; svz, subventricular zone; vz, ventricular zone. For other abbreviations see list*.

^*^APHcl is referred as dorsolateral corticoid area or CDL by the followers of the conclusions of the avian brain nomenclature forum (Reiner et al., 2004; see also Atoji and Wild, 2005). Herein we use APHcl as a preferred term to emphasize its relation to other APH subdivisions (see also Redies et al., 2001; Puelles et al., 2007).

^*^^1^DLP is referred as caudal dorsolateral pallium (CDL) in the atlas of the chick brain, by Puelles et al. (2007), and was previously included as part of the avian temporo-parieto-occipital area or TPO (see Discussion in Abellán et al., 2009).

### Gene expression patterns with respect to major pallial subdivisions in mouse and chicken

The mRNA expression patterns of all genes analyzed were largely conserved between mouse and chicken, although some differences were also appreciated. During very early development (E11.5 in mouse; E6-E7 in chicken), *Lef1* was strongly and distinctly expressed in the ventricular zone (vz) of the medial pallium (MP) and the pallial septum (PSe) (chicken: **Figures 6A,B**), although it also showed generally weak expression in the vz of other pallial sectors. By E13.5 in mouse (Figures 1A,B,D,E) and E8 in chicken (**Figures 6C**, **7A**), *Lef1* became primarily restricted to the vz of the medial pallium and pallial septum (Tables 1A, 2A).

At E13.5–E15.5 in mouse (Figures 1–**3**) and E8-E9 in chicken (**Figures 6**, **7**), the medial pallial sector was characterized by strong or moderate vz expression of *Lef1* (Figures 1, **3**, **6C**, **7A**)*, Lhx2* (Figures 2A–D, **6D,E**), and *Lhx9* (Figures 2E–K, **6F**, **7B,C**), but not *Lhx5* (Figures 1C, **6G**). This feature allowed the distinction of the medial pallium from other progenitor pallial sectors, such as: the pallial septum (PSe), expressing *Lef1* (Figures 1A, **6B**), *Lhx2* (**Figure 6E**), *Lhx9* (**Figure 6F**), and also *Lhx5* (Figures 1C, **6G**) in the vz; the dorsal pallium (DP), expressing strongly *Lhx2* in the vz (Figures 2A–D, **6D**), but not *Lef1* (except its medialmost, cingulate-related area; Figures 1A,B,D), *Lhx9* (Figures 2E–G) or *Lhx5* (Figure 1C) (data on *Lhx2, Lhx9*, and *Lhx5* in chicken DP is published in Abellán et al., 2009, 2010); and the lateral (LP) and ventral (VP) pallia, showing generally weak expression of *Lhx2* in the vz (Figures 2A, **6D,E**), but no vz expression of *Lef1* (Figures 1A,B, **6C**) nor *Lhx5* (Figures 1C, **6G**) (summarized in Tables 1, 2; for the lateral pallial sector, we followed a recent redefinition done by Puelles, 2014).

Based on the combinatorial gene expression patterns studied here and on published data (Puelles et al., 2000, 2007; Medina et al., 2004; Abellán et al., 2009; Puelles, 2014), we tentatively distinguished two new pallial sectors, which we named the dorsolateral caudal pallium (DLP) and the ventrolateral caudal pallium (VLP) (Tables 1, 2). The DLP was previously described in chicken as a distinct subdivision belonging to either the lateral pallium (Puelles et al., 2007) or ventral pallium (Abellán et al., 2009), and has been called temporo-parieto-occipital area or pallium externum in some studies (for example, Veenman et al., 1995; Atoji and Wild, 2005). In contrast to the dorsal and lateral pallia, the DLP expressed *Lhx9* in the mantle throughout development (**Figures 6F**, **10A,H,I**) and, for this reason, was previously suggested to be part of the ventral pallium (Abellán et al., 2009). However, in contrast to the ventral pallium, the DLP showed abundant vz/mantle expression of *Emx1* (chicken: named CDLx in Figure 10 n of Puelles et al., 2000) and *Lhx2* (**Figure 10B**). In addition, in contrast to the medial pallium, the DLP did not express *Lef1* (**Figure 6C**, Table 2). Herein, we tentatively identified a comparable pallial subdivision in the mouse, giving rise to the lateral entorhinal cortex (LEnt), with similar topological position and genetic profile [no expression of *Lef1* in vz. (Figures 1E, 3F,G), but expressing *Lhx9* in the mantle (Figure 2H) and *Emx1* in vz/mantle (see Allen Developing Mouse Brain Atlas); Table 1]. On the other hand, the VLP [for the moment only identified in chicken, and giving rise to the arcopallium (A)] differed from the ventral pallium [giving rise to the nidopallium (N) and piriform cortex (Pir)] for its strong expression of *Lhx9* (**Figures 7B,C**, **9E**, **10D,F,J**; Abellán et al., 2009), *Lhx2* (**Figures 7D**, **10E**), and *Emx1* (see Figure 10p in Puelles et al., 2000) in both the ventricular zone and mantle (Table 2).

In addition to the expression in the vz, most derivatives of the medial pallium of mouse and chicken also showed moderate to strong expression of *Lef1* and *Lhx2* at least during early and intermediate developmental stages, while some or many of them also expressed *Lhx9* (see details in next sections; Tables 1, 2). These features, linked to the molecular identity of the medial pallial vz, helped to identify and compare the medial pallial derivatives between mouse and chicken. The results on the combinatorial expression of *Lef1* and other developmental regulatory genes in the developing hippocampal complex (including hippocampal formation and entorhinal cortex) are explained in detail below, first for the mouse (Figures 1–**5**) and then for the chicken (**Figures 6**–**11**).

### Combinatorial expression of *Lef1, Lhx2, Lhx9, Lhx5, Lmo3*, and *Lmo4* in the developing hippocampal complex of mouse

#### E12.5–E13.5

During early development, the medial pallial sector (MP) of mouse was distinguished by its moderate to strong expression of *Lef1, Lhx2*, and *Lhx9* in the vz and in postmitotic cells migrating into the mantle (Table 1, Figures 1, 2). Outside the medial pallium, the only additional pallial sector expressing *Lef1* in the vz was the pallial septum (PSe) and the adjacent part of the dorsal pallium [DP; cingulate part of neocortical primordium (Cg)] (Table 1A, Figure 1A). Based on the expression of *Lef1* (Figures 1B,D,E)*, Lhx2* (Figures 2A–D), and *Lhx9* (Figures 2E–K), the medial pallium appeared to include the progenitor zones of the *indusium griseum* (rostrally; IG neuroepithelium, ign), the dentate gyrus (DG neuroepithelium, dgn), the CA fields (CA neuropeithelium, can), the subiculum (S neuroepithelium, sn), at least part of the amygdalo-hippocampal transition area (AHi in Figure S1), and a medial and caudal part of the entorhinal cortex (corresponding to the primordium of the so-called medial entorhinal cortex; MEnt). In contrast, the lateral entorhinal cortex (LEnt) appeared to derive from a distinct pallial sector, the DLP, which vz did not express *Lef1* (Figure 1E) or *Lhx9* (Figure 2H). Nevertheless, the lateral entorhinal cortical plate showed moderate *Lef1* expression (Figure 1E), resembling the adjacent part of the neocortex cortical plate.

#### E15.5–E16.5

*Lef1, Lhx2*, and *Lhx9* continued to be expressed in the vz and mantle of the mouse medial pallium during intermediate development [Table 1B, Figures 3, 4; data on *Lhx2* at E15.5 is available in the Allen Developing Mouse Brain Atlas and published elsewhere (Bulchand et al., 2003); for comparative reasons, such data is included in Table 1B]. The expression of *Lef1* was moderate to strong in all medial pallial-derived areas [*indusium griseum* (IG), dentate gyrus (DG), CA fields, subiculum (S), medial entorhinal cortex (MEnt); Figure 3], while *Lhx9* remained moderate to strong in most of them but started to be downregulated in the developing CA1 field and, especially, in the developing subiculum (Figures 4A–C).

During intermediate development, medial pallial derivatives also showed expression of *Lmo4* and *Lmo3*, with different patterns (Table 1B, Figure 4). *Lmo4* showed moderate to strong expression in the developing CA fields, subiculum, and medial entorhinal cortex, while the developing *indusium griseum* and dentate gyrus only showed weak or weak to moderate *Lmo4* expression, respectively (Figures 4D–G). On the other hand, *Lmo3* showed weak or moderate expression in the developing CA1, subiculum, and medial entorhinal cortex, but was not expressed in the developing CA3, dentate gyrus and *indusium griseum* (Figures 4H–J).

At E15.5–E16.5, while the medial entorhinal cortex (MEnt) showed gene expression patterns highly similar to those in other medial pallial derivatives, the lateral entorhinal cortex (LEnt, a DLP derivative) differed in the expression of *Lef1* (Figures 3F,G), *Lhx9* (Figure 4K), *Lmo4* (Figure 4G), and *Lmo3* (Figures 4I,J; see also Table 1B). For example, in the DLP/LEnt, expression of *Lef1* (Figures 3F,G) and *Lhx9* (Figure 4K) was only weak or moderate, and restricted to the mantle. Moreover, in the LEnt, expression of *Lmo4* was only weak to moderate (Figure 4G), while *Lmo3* showed a bi-layered expression pattern (apparently superficial and deep to the lamina dissecans or layer IV), making it different from the MEnt (Figures 4I,J).

#### E17.5-P0

During prenatal stages, the expression of *Lef1* became weak in most of the medial pallium, and almost disappeared in the CA3 field, with the only exception of the dentate gyrus (DG), where it remained moderate to strong (Figures 5A–D). In contrast, the lateral entorhinal cortex retained moderate expression of *Lef1*.

At perinatal stages, *Lhx2* (Figures 5E–E″)*, Lmo3* (Figure 5F), and *Lmo4* (Figure 5G) intensified their expression in the pallium, but retained the specific patterns observed before for the different pallial divisions and subdivisions (Figures 5E–G). The expression of *Lhx2* was strong or very strong in most subdivisions of the medial pallium (including vz and mantle; Figures 5E–E″), except the *indusium griseum*, where the expression was moderate. *Lmo3* showed very strong expression in the principal cell layer of the subiculum (S) and CA1, and moderate expression in the *indusium griseum* (IG) and medial entorhinal cortex (MEnt), but its expression was very weak in CA3 and absent in the dentate gyrus (DG) (Figure 5F). *Lmo4* expression was moderate to strong in most medial pallial subdivisions (with the pyramidal cell layer of CA1 showing the strongest expression), except the *indusium griseum* and the dentate gyrus, which showed only weak expression (Figure 5G). On the other hand, *Lhx9* was moderate to strongly expressed in the *indusium griseum*, dentate gyrus, CA3 field, and medial entorhinal cortex, but appeared completely downregulated in the CA1 and subiculum at E18.5.

Regarding the lateral entorhinal cortex, at perinatal stages continued showing weak to moderate expression of *Lhx2* (Figures 5E,E′), *Lhx9*, and *Lmo3* (Figure 5F), as during previous stages. In contrast, expression of *Lmo4* became strong at these stages (Figure 5G).

### Combinatorial expression of *cLef1, cLhx2, cLhx9, cLhx5, cProx1, cLmo3*, and *cLmo4* in the developing hippocampal complex of chicken

#### E8

Similarly to the mouse, at E8 the medial pallial sector (MP) of chicken embryos could be distinguished by its moderate to strong expression of *cLef1* (Figures 6C, 7A)*, cLhx2* (Figures 6D,E, 7D), and *cLhx9* (Figures 6F, 7B,C) in the vz (Table 2A). The rest of the pallium did not express *cLef1* in the vz at E8, and the different pallial subdivisions [dorsal (DP), dorsolateral-caudal (DLP), lateral (LP), ventral (VP), ventrolateral-caudal (VLP)] could additionally be distinguished by a region-specific combinatorial expression of *cLhx2, cLhx9, cLmo3*, and *cLmo4* in the vz, subventricular zone (svz, identified based on Charvet et al., 2009), and/or mantle zones (Table 2A).

Based on the expression of *cLef1* (Figures 6C, 7A)*, cLhx2* (Figures 6D,E, 7E), and *cLhx9* (Figures 6F, 7B,C) at the vz, it appeared that the medial pallium in chicken included the progenitor zones of the hippocampus (including the V-shaped area) and the parahippocampal areas (APH), including its caudolateral part (APHcl), which is referred as dorsolateral corticoid area or CDL by some authors (see Discussion; Table 2A). At caudal levels, the avian medial pallium also appeared to include the progenitor zone of the so-called entorhinal cortex (Ent) and the amygdalohippocampal area (AHi, at least its transition part, as defined by Puelles et al., 2007) (Figures 7A,B). Regarding other genes, the chicken medial pallium also showed moderate to strong expression of *cLmo4* in the mantle (Figures 7E,H; Table 2A), and expression of *cLmo3* in the intermediate zone (a mantle part near the vz, possibly containing migratory neuroblasts) in the APH, with an increasing gradient toward caudolateral levels (Figures 7F,G).

#### E10–E14

During these stages, the medial pallium continued to show distinct expression of *cLef1, cLhx2*, and *cLhx9*, with patterns similar to those found previously (Figures 8–**10**; Table 2B). As in previous stages, at E10–E14 *cLef1* was expressed in the vz of the medial pallium (MP), although only in part of it because it was downregulated in the vz of APHl, APHcl, and entorhinal cortex (Ent) (Figures 8C,D,G,G′). In addition to the vz, the expression of *cLef1* now extended into the medial pallial mantle (Figures 8A–D,G,G′; Table 2B). The expression of *cLef1* was moderate to strong in the hippocampus (V-shaped area, here named dentate gyrus or DG, as explained below) and the different APH subdivisions, except the APHcl and the Ent where *cLef1* expression was only weak (Figures 8A–D,G,G′; Table 2B). The expression of *cLef1* allowed distinction of a novel subdivision, called by us the rostral APH (APHr), which showed very strong expression (Figures 8A–D). The APHr may correspond or include the apical part of APH described in the chick brain atlas by Puelles et al. (2007). Comparison of *cLef1* with radial glial fiber disposition suggested that APHr vz occupied the rostralmost pole of APH as seen in frontal section (Figure 8C); a group of *cLef1*-expressing cells appeared to separate from this rostral location, suggesting that they migrated tangentially toward gradually more distant superficial, dorsomedial and caudal positions; we called this migrated part ectopic APHr or APHre (Figures 8B,D,G,I). At intermediate and caudal levels, this extension of APHr (APHre) occupied a small and distinct superficial area at the surface of APHm (Figures 8D,G; compare Figures 8G,I), which appeared to correspond to the parvocellular hippocampal area identified by Atoji and Wild in adult pigeons (2004). During intermediate developmental stages, *cLef1* started to be expressed in restricted parts of the mantle of both the dorsal pallium (hyperpallium, H) and the lateral pallium (mesopallium, M) (Figures 8A–C; see also Figure S2).

*cLhx2, cLhx9, cProx1, cLmo3*, and *cLmo4* were also expressed in the mantle of the chicken medial pallium at E10–E14, but showed differences between distinct subdivisions (Figures 8–**10**; Table 2B). In particular, *cProx1* was exclusively expressed in a large part of the so-called avian hippocampus, including a large part of the dorsal hippocampus or V-shaped area (dentate gyrus primordium and hippocampal sector 1 or Hi1 of Puelles et al., 2007) and the so-called ventral hippocampus (Figures 8E,H; Table 2B); this makes this chicken medial pallial subdivision comparable by position, embryonic origin, and molecular profile to the mouse dentate gyrus or DG, and we called it accordingly. On the other hand, *cLmo4* (Figures 9A–C,H,K,N, 10G) and *cLhx2* (Figures 10B,C,E) were moderate to strongly expressed in the whole mantle of all subdivisions of the medial pallium, with the strongest signal levels observed in APHr, APHm, and APHi. In contrast, *cLhx9* and *cLmo3* expressions in the mantle were restricted to different subdivisions. Thus, *cLhx9* was expressed weakly in APHl and strongly in APHcl and entorhinal cortex (Figures 9D,E, 10D,F,H,I). *cLmo3* showed generally weak expression in DG, and the superficial layer of APHr, APHm, APHi, and APHl subdivisions (Figures 9F,G,I,J,L,M).

#### E16–E18 and hatchlings

While the expression of *cLhx2* remained moderate to strong in the vz and mantle of the whole medial pallium, *cLef1* and *cLhx9* were completely or almost completely downregulated in the medial pallial vz, and their expression became restricted to only parts of the mantle (Figure 11). *cLhx9* retained its expression in the mantle of APHl, APHcl, and entorhinal cortex (not shown here, but seen in Figures 5, 6 in Abellán et al., 2009). *cLef1* became downregulated in most medial pallial areas but retained a very strong expression in APHr and its ectopic extension (APHre), which was still visible at P2 (Figures 11H,I). On the other hand, *cProx1* retained its distinctive expression in DG at least until P2 (Figure 11J; Figure S2). Finally, the expression patterns of *cLmo3* and *cLmo4* in the chicken medial pallium during pre-hatching stages were similar to those seen before (E12–E14). By E18, *cLmo3* expression was weak in DG and Ent, but moderate in parts of most APH subdivisions, except APHl, where it was strong (Figures 11A–C). The expression pattern of *cLmo3* in DG and APH was still similar by P0. In APHi, *cLmo3* expression was located deep and superficial to the principal cell layer. However, in APHl and medial APHcl the expression was ample but left empty, free of expression, patches or islands of the cortical plate. By P0, *cLmo3* expression became moderate in the entorhinal cortex. On the other hand, at E16–E18, the expression of *cLmo4* was moderate to very strong in all medial pallial subdivisions, being remarkable in parts of APH (Figures 11D–F). By P0, *cLmo4* expression still was remarkably strong in APHm, APHi, and the ectopic part of APHr (Figure 11G). However, the expression became weak in DG.

## Discussion

### Comparison of the hippocampal formation of mouse and chicken based on combinatorial gene expression patterns

In this study, we used the combinatorial expression patterns of seven developmental regulatory genes (*Lef1, Lhx2, Lhx9, Lhx5, Lmo3, Lmo4*, and *Prox1*), together with analysis of topological position and published data on these or other genes, to identify the medial pallial derivatives and define its major subdivisions in mouse and chicken, and to compare such subdivisions between both species. In both mouse and chicken, the medial pallium is defined as a pallial sector adjacent to the cortical hem (expressing *cLhx5* and *cWn8b*; Figures 6G, 8F) and roof plate/choroid tela (expressing *cWnt8b*, Figure 8F), showing moderate to strong vz expression of *Lef1, Lhx2*, and *Lhx9*, but not *Lhx5*, at least during early developmental stages. The topological position and the combination of genes at early developmental stages make the medial pallium different from other neighboring pallial sectors, such as the pallial septum (which additionally expresses *Lhx5*), the dorsal pallium (which does not express *Lhx9* in the vz, and is mostly devoid of *Lef1* except its medialmost, cingulate/retrosplenial areas), the dorsolateral caudal pallium (which does not express *Lef1* nor *Lhx9* in the vz), and the ventral/ventrolateral-caudal pallia (which do not express *Lef1* in the vz). Based on this gene combination, often also present in the mantle, we propose that the *indusium griseum*, the hippocampal formation (DG, CA fields, and subiculum), the medial entorhinal cortex, and part of the amygdalo-hippocampal transition area of mouse are medial pallial derivatives. It is likely that the presubiculum and parasubiculum also derive from the medial pallium (see these areas expressing *Lhx2* in Figure 5E″), although our data were insufficient to clearly determine expression of *Lef1* in these areas. In the chicken, based on the same position and gene combination, we propose that the hippocampus (including the V-shaped area and the ventral hippocampus), the APH (including its caudolateral part, often called CDL; Reiner et al., 2004; Atoji and Wild, 2005), the entorhinal cortex, and the amygdalo-hippocampal transition area are medial pallial derivatives.

The genes *Lhx2, Lhx9*, and *Lef1* were previously described to be expressed in the developing hippocampal formation of mouse (*Lhx2*: Porter et al., 1997; Bulchand et al., 2001; Monuki et al., 2001; Vyas et al., 2003; *Lhx9*: Rétaux et al., 1999; Vyas et al., 2003; Abellán et al., 2009; *Lef1*: Galceran et al., 2000; Choe et al., 2013). Herein, we provide more details on their expression in other medial pallial derivatives, such as the *indusium griseum*, part of the amygdalo-hippocampal transition area and the medial entorhinal cortex. The common origin with other parts of the hippocampal formation may explain some of their similar features and connections (see discussion for the entorhinal cortex below).

The present study is the first one that uses the three genes in combination, in a comparative context and in a comprehensive way, for trying to identify the medial pallial derivatives in the chicken. There are previous, separate reports of expression of these genes in the developing medial pallium of chicken, but none of these showed enough detail (*cLhx2* and *cLhx9*: Abellán et al., 2009; this study was centered in the ventral pallium; see also data of *Lhx9* in the zebra finch: Chen et al., 2013) and/or signal quality (*cLef1*: Gupta et al., 2012). Based on the combinatorial expression patterns presented here, the chicken medial pallium is larger than previously thought since it includes not only the hippocampus (including the V-shaped area and the ventral hippocampus) and medial parts of APH (our APHm, APHi, APHl; simply named APH in the proposal of the Avian Brain Nomenclature Forum; Reiner et al., 2004), but also the caudolateral part of APH, the entorhinal cortex, and the amygdalo-hippocampal transition area. The caudolateral APH (APHcl, using the nomenclature of Redies et al., 2001; Puelles et al., 2007) is called the dorsolateral corticoid area by many authors (CDL; Reiner et al., 2004; Atoji and Wild, 2005). Its medial pallial origin possibly explains its three-layered cytoarchitecture similar to other APH areas (Redies et al., 2001; Puelles et al., 2007), and its extensive connections with other parts of the APH, as shown in pigeons (Atoji and Wild, 2005). Based on its connections, Atoji and Wild (2005) proposed that the APHcl/CDL is comparable to the cingulate cortex of mammals, which primordium also expresses *Lef1* during development (present data). However, while the APHcl/CDL derives from the medial pallium (having vz expression of *Lef1, Lhx2*, and *Lhx9*), the cingulate cortex and other parts of the neocortex derive from the dorsal pallium (showing lack of expression of *Lhx9* at the vz), which disfavors the homology of these two structures. Our data support that APHcl/CDL is really a medial pallial derivative and, as such, part of the avian hippocampal complex; therefore, we recommend to call it simply APHcl and to abandon the term CDL, which is confusing because it is also employed by some authors to refer to the DLP (see, for example, Puelles et al., 2007; Belgard et al., 2013). In addition, we found a novel cell group, the APHr (maybe comparable to the apical APH of Puelles et al., 2007), which could be distinguished by its intense expression of *Lef1* from E10 onwards. While *Lef1* started to be downregulated in most of the medial pallium, its expression was intensified in APHr during intermediate and late embryonic development, and was still seen defining this cell group after hatching (P2). As discussed later, an ectopic migrated part of APHr appears to reach intermediate and caudal hippocampal formation levels, where it appears to correspond to the so-called parvocellular region of Atoji and Wild (2005).

The present data agree with previous claims of homology of the avian hippocampus and APH with the hippocampal formation of mammals (reviews in Dubbeldam, 1998; Reiner et al., 2004; Striedter, 2005; Atoji and Wild, 2006; Papp et al., 2007), which were based on identical topological position and embryological origin (Ariens-Kapper et al., 1936; Källén, 1962; Redies et al., 2001; Puelles et al., 2007), and some similarities in cyto- and chemo-architecture (Erichsen et al., 1991; Montagnese et al., 1996; Tömböl et al., 2000; see also Herold et al., 2014), connections (Benowitz and Karten, 1976; Casini et al., 1986; Székely and Krebs, 1996; Székely, 1999; Atoji et al., 2002; Atoji and Wild, 2004), and function (Bingman et al., 1984, 2003; Sherry et al., 1992; Colombo and Broadbent, 2000; Clayton et al., 2003; Mayer et al., 2013). Similarly to that of mammals, the avian hippocampal formation is involved in episodic and spatial memory, and contains location-specific and other types of cells involved in spatial navigation (Clayton et al., 2003; Bingman and Sharp, 2006). It also shows oscillatory activity similar to the theta rhythms (Siegel et al., 2000), LTP and LTD synaptic plasticity involved in learning and memory, synaptic modification after training, and evidence of adult neurogenesis (reviewed by Papp et al., 2007). Crucial for the argument of homology is that the hippocampal formation has also been identified in reptiles, and was likely present in the common ancestor of amniotes (reviewed by Rodríguez et al., 2002; Papp et al., 2007; Medina and Abellán, 2009).

Our data also agree with more recent proposals of homology based on massive gene expression data in the adult pallium, which show a striking similarity of mouse and chicken medial pallial derivatives regarding their gene expression profile (for example, Belgard et al., 2013). However, some recent studies have revealed that the hippocampal formation of different avian species also shows a genetic expression profile similar to that of the arcopallium during development (Chen et al., 2013) and in the adult (Jarvis et al., 2013). This particularly refers to the expression of the transcription factors Lhx9 and ER81. However, *Lef1*, which in mammals has been shown to be essential for the development of the hippocampal formation (see above), is expressed in the developing hippocampal formation of chicken (E8–E9), but not in the arcopallium. After E10, *Lef1* also starts to be expressed in parts of the mantle of the chicken dorsal pallium (hyperpallium) and lateral pallium (mesopallium). It is important to remember that most developmental regulatory genes are expressed in more than one region; for example, this is so for *Emx1, Emx2, Pax6, Lhx2, Lhx9*, and *ER81*, expressed in several pallial subdivisions, but some also in the subpallium and outside the telencephalon, and even outside the nervous system (Puelles et al., 2000; Abellán and Medina, 2009; Abellán et al., 2009; Tzchori et al., 2009; Chen et al., 2013; present data). Their function is region, time, and context dependent. This also applies to Lef1, which is expressed in the brain and other tissues, in complex patterns that change throughout embryonic development and postnatally (present data; Oosterwegel et al., 1993; Nagalski et al., 2013), having roles that are context-dependent (Mao and Byers, 2011). The context relies on the molecular networks present in the tissue, which change between regions and with time. The molecular network present in the tissue at any time is essential for understanding both the interactions between transcription factors or other regulatory proteins and their region- and time-specific function. For this reason, we pay special attention to both the topological position (Nieuwenhuys, 1998, 2009; Striedter, 2005) and the combinatorial expression patterns of regulatory genes seen during early development (see also discussion in Puelles and Medina, 2002; Puelles and Ferran, 2012; Medina et al., 2013). Studies using knockout mice have shown that Lef1 is one of the key actors involved at early stages in the development of the hippocampal formation, but this transcription factor acts in combination with other regulatory proteins, such as Wnt and BMP proteins, produced at the cortical hem and/or roof plate (Galceran et al., 2000; Choe et al., 2013). How Lef1 interacts with Lhx2 (also essential for hippocampal development; Bulchand et al., 2001; Vyas et al., 2003), Lhx9, and ER81 during medial pallial development is unknown. The role of Lef1 in the development of other brain regions outside the medial pallium (such as the hyperpallium/dorsal pallium or the thalamus) is also unknown. Due to its far lateroventral position, the arcopallium appears to be out of the effect of roof plate/cortical hem BMP/Wnt signals (if existent, such effect is likely very weak; see also Medina and Abellán, 2009; Aboitiz and Zamorano, 2013). A partially different network of transcription factors (without the implication of Lef1) is important for arcopallial development (such as Lhx9, ER81, and other), although the hierarchy, interactions and functions of the different factors within the network are still unknown.

### Hippocampal formation subdivisions in mouse and chicken

**Figure 12:**
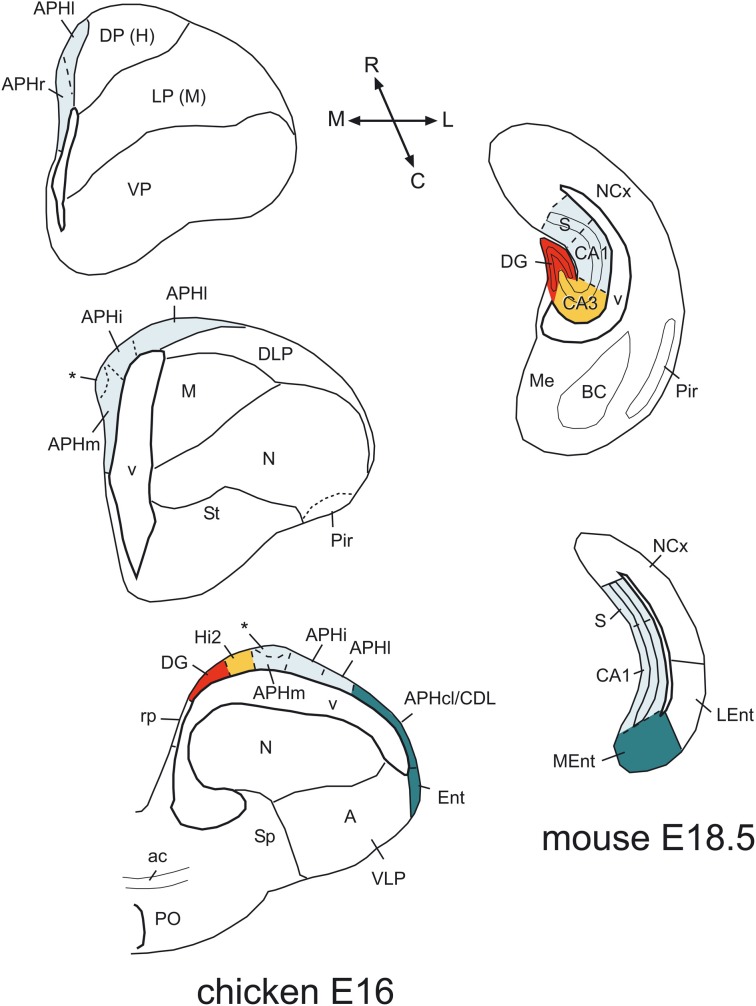
**Comparison of chicken and mouse medial pallial subdivisions.** Schematic drawings of frontal sections through the telencephalon of a chicken (at E16) and a mouse (at E18.5), at rostral intermediate, or caudal levels, showing the major subdivisions of the medial pallium. A color code is used to compare these subdivisions between species. In these schemes, dorsal is to the top and medial is to the left. In the chicken, the rostralmost part is represented by the APHr. The asterisk points to an ectopic part of chicken APHr (possibly a group tangentially migrated cells), observed at the surface of APHm at intermediate and caudal levels of the medial pallium. The rostralmost part of mouse is not represented here, but appears to include the indusium griseum. For abbreviations see list. See text for more details.

The combinatorial expression of *Lef1, Lhx2, Lhx9, Prox1, Lmo4*, and *Lmo3* was useful for defining some molecular features of the major subdivisions of the hippocampal formation, and for comparative purposes. Below we discuss the evidence suggesting the comparison of specific chicken subdivisions with the mammallian DG/CA3 and the CA1/subiculum (Figure 12).

#### Dentate gyrus and CA3

The mouse DG, occupying the medialmost topological position within the medial pallium, typically showed moderate to strong expression of *Lef1, Lhx2, Lhx9*, and *Prox1*. Of these, *Lef1* and *Prox1* have been shown to be of crucial importance. Lack of Lef1 function in mouse leads to agenesis of the DG (Galceran et al., 2000), while conditional inactivation of Prox1 in mouse showed that this transcription factor is essential for specification and maturation of DG granule cells, and maintenance of their cell identity throughout life (Lavado et al., 2010; Karalay et al., 2011; Iwano et al., 2012). In the DG of adult mice, *Prox1* is also involved in intermediate progenitor maintenance and maturation of new granule cells (Lavado et al., 2010; Karalay et al., 2011).

In contrasts, there is much controversy on the location or existence of a DG in birds and reptiles. Based on different data, there are diverse opinions on possible areas homologous to DG in birds: V-shaped area or part of it, vs. part of APH (for example, Montagnese et al., 1996; Székely, 1999; Atoji et al., 2002; Atoji and Wild, 2004; Suárez et al., 2006; Puelles et al., 2007; Herold et al., 2014). Moreover, some authors claim that DG may be a novel acquisition of mammals (Papp et al., 2007), which would imply that there is no homolog in birds. However, our data on *Lef1* and *Prox1* strongly suggest that a large part of the so-called avian hippocampus, including its dorsal (the part of V-shaped area encompassing the dentate gyrus primordium and hippocampal sector 1 of Puelles et al., 2007) and ventral parts (Atoji et al., 2002), could be homologous to mammalian DG, if confirmed its presence in reptiles. Gupta et al. (2012) reached a similar conclusion based on *Prox1* in V-shaped area during early/intermediate development (E8–E14), although these authors included the whole V-shaped area and did not mention the ventral hippocampus. Our data show that the dorsalmost part of V-shaped area (hippocampal sector 2 or Hi2 of Puelles et al., 2007; Table 2B) does not express *Prox1* at E10–E12 or later (Figures 8H, 11J), raising doubts on the homology of this dorsal part. Gupta et al. (2012) showed that chicken DG cells are born between E6 (the majority) and E10 from the vz deep to the V-shaped area, and start to express Prox1 4 days later (from E8 on). Our data show that *Prox1* continues to be expressed in chicken DG after hatching (at least until P2), but *Lef1* is downregulated, similarly to the findings in mouse (Nagalski et al., 2013).

Regarding CA3, in mouse this area shares some features with DG, such as lack of *Lmo3* expression (see Table 1B), but it does not express *Prox1*. In chicken, the dorsal part of V-shaped area, with no expression of *Prox1*, does not express *Lmo3* either, and may be comparable to CA3 (asterisks in Figures 11B,C). Curiously, following postmitotic inactivation of *Prox1* in mouse, immature neurons of DG lose their granule cell identity and differentiate into CA3 pyramidal neurons (Iwano et al., 2012). This means that DG immature neurons have the potential of becoming either granule cells or CA3 pyramidal cells. Moreover, recent data have shown that, in rats, CA3 field includes a subpopulation of granule cells, which contain calbindin and Prox1 as those of DG (Szabadics et al., 2010). These interesting observations have implications for understanding hippocampal evolution, since perhaps both DG and CA3 evolved from a common field, which splits into two separate fields either by downregulation of *Prox1* in one part (the CA3) or by novel expression of *Prox1* in one of the parts (the DG). To know what was the ancestral situation in amniotes, it is mandatory to study *Lef1* and *Prox1* in different reptiles, including lizards and other Squamates, which are currently considered a sister group of Archosauria (birds, crocodiles and perhaps turtles) and, as such, excellent models for understanding the basal condition in sauropsids (Zardoya and Meyer, 1998; Meyer and Zardoya, 2003; Fong et al., 2012). Comparison of the chicken, crocodile, and lizard hippocampal formation (Nissl images in Papp et al., 2007, for lizard and crocodile; Puelles et al., 2007, for chicken) points to the striking topological and cytoarchitectonic similarity of the chicken ventral hippocampus and the lizard/crocodile medial cortex, and the chicken V-shaped area (especially its dorsal part) to the lizard/crocodile dorsomedial cortex. Although some authors have suggested that the reptilian medial cortex is comparable to mammalian DG and the reptilian dorsomedial cortex is comparable to CA3 (Martínez-Guijarro et al., 1990), other authors suggested that the reptilian medial cortex is comparable to the mammalian *indusium griseum* (Künzle, 2004), or that both reptilian cortices maybe like mammalian CA3 (Papp et al., 2007). The possible common origin of DG and CA3 may explain why the connections of the avian V-shaped area and the reptilian medial/dorsomedial cortices are a mixture of those of mammalian DG and CA3 [reciprocal connections with the septum, and both ipsi- and contralateral (commissural) projections to other parts of the hippocampal formation; birds: (Casini et al., 1986; Atoji and Wild, 2004; Montagnese et al., 2004); for mammals see (Witter and Amaral, 2004); reptiles: (Lopez-Garcia and Martinez-Guijarro, 1988; Olucha et al., 1988; Martínez-Guijarro et al., 1990; Hoogland and Vermeulen-VanderZee, 1993)]. In any case, it is clear that the avian hippocampal formation has undergone partial divergence during the hundreds of millions of years of separate evolution (Striedter, 2005), which explains why some of the hippocampal subdivisions and features found in extant birds do not really fit well with any of those found in reptiles or mammals (see also Papp et al., 2007; Herold et al., 2014).

In addition to its role in DG granule cell specification, differentiation, and survival (reviewed by Karalay and Jessberger, 2011), recent data in mouse showed that, from late embryonic stages, *Prox1* is also expressed in subsets of neocortical and hippocampal interneurons, which derive from the caudolateral ganglionic eminence and the preoptic area of the subpallium (Rubin and Kessaris, 2013). However, our data in chicken did not allow to discriminate the presence of *Prox1*-expressing interneurons.

#### CA1 and subiculum

During early (chicken) and/or intermediate (mouse and chicken) development, in addition to *Lef1* and *Lhx2*, most medial pallial derivatives also show moderate to strong expression of *Lmo4*, while some of them (including CA1 and subiculum) also show *Lmo3* expression in an area- and layer-specific way. In the mouse, the strongest expression of *Lmo4* occurs in the CA1, while the strongest expression of *Lmo3* is seen in the subiculum. In the chicken, the strongest *Lmo4* expression is seen in APHm and APHi [roughly corresponding to the dorsomedial APH sector (DM) of Atoji and Wild, 2004], while the strongest *Lmo3* expression is seen in APHl [corresponding to the dorsolateral APH sector (DL) of Atoji and Wild, 2004; for comparison see Suárez et al., 2006]. These subdivisions show associational connections with other hippocampal areas, as well as descending projections to the septum, the nucleus accumbens, the pallial amygdala, the extended amygdala, and the hypothalamus, including the mammillary region (Atoji et al., 2002, 2006; Atoji and Wild, 2004, 2005). Importantly, the APHm,i,l (DM and DL fields) are extensively and reciprocally connected with the DG/CA3 area (V-shaped area) (Atoji et al., 2002), thus establishing the basis for the recurrent, associational architecture typical of the hippocampal formation in mammals, and needed for memory acquisition (Papp et al., 2007). Based on their topological position, embryonic origin, genetic profile, and connectivity patterns, these APH subdivisions together appear comparable to the CA1/subiculum of mammals (see also Atoji and Wild, 2004; Suárez et al., 2006).

### Radial vs. tangential cell migrations within the medial pallium: the cases of the APHr and DG

Data in chicken and in different mammalian species show that the majority of the neurons of the hippocampal formation migrate radially (following radial glial fibers) from the medial pallium neuroepithelium (mammals: Eckenhoff and Rakic, 1984; Rickmann et al., 1987; Altman and Bayer, 1990a,b; Li and Pleasure, 2005; chicken, Gupta et al., 2012). The exception to this rule is the case of the GABAergic interneurons that populate the hippocampal formation, which migrate from the subpallium (Pleasure et al., 2000; Cobos et al., 2001). In addition, in chicken a part of the cells of APHr (the ectopic APHr or APHre) appears to migrate tangentially within the medial pallium to occupy more caudal, dorsomedial, and superficial positions (present results). Based on *Lef1* expression and radial glial fiber disposition, the APHr vz appears to be located at very rostral APH levels (maybe corresponding to the apical APH of Puelles et al., 2007), where *Lef1* occupies the whole mantle (Figure 8C; Figure S2A). A band of *Lef1*-expressing cells (the APHre) appears to extend from this origin, and progressively occupies more superficial, dorsolateral and caudal positions. At intermediate and caudal levels, the *Lef1* expression domain related to APHre lies at the surface of APHm (Figures 8D, 9J, 11G) and appears to correspond to the so-called parvocellular region of the hippocampal formation (Atoji and Wild, 2005). Thus, this observation suggests that the neurons of the parvocellular region arrive at their final destination by tangential migration. Supporting this proposal, this region is avoided by radial glial fibers that produce the underlying APHm (Figure 8I). The relation of APHr/APHre to other hippocampal subdivisions of chicken or other amniotes and the function of this cell group remain unknown.

In mammals, the DG granule neurons follow a special type of radial migration due to deformation of the radial glial fibers at the medialmost pallial edge, during the pallial growth that occurs in later developmental stages (Eckenhoff and Rakic, 1984; Rickmann et al., 1987; Li and Pleasure, 2005; note that some authors do not consider this migration to be radial: Altman and Bayer, 1990a). Such deformation of the radial glial fibers is not visible in the medial pallium of chicken (Gupta et al., 2012; present results of radial glial fibers), possibly because it does not grow as much as in mammals.

### Entorhinal cortex: two divisions, two embryonic origins

Our data in mouse suggest that the two major divisions described in the entorhinal cortex of different mammals originate in separate pallial domains, the MEnt (caudomedially located) from the medial pallium, and the LEnt (rostrolaterally located) from the dorsolateral caudal pallium (Figure 12). In particular, based on the combinatorial expression of *Lef1, Lhx2, Lhx9*, and *Lmo4*, the MEnt appears to derive from the same embryonic domain that produces the hippocampal formation. This may explain some of the distinct features found in MEnt (but not LEnt) (Sewards and Sewards, 2003), such as the presence of cells involved in processing spatial cues (grid cells, head-direction cells, and border cells, which respond to specific position, direction and orientation, and are able to precisely map the spatial environment), and its implication in transmitting information on the spatial context of an experience to the hippocampal formation (Leutgeb et al., 2005; Knierim et al., 2013; Zhang et al., 2013). Moreover, lesion experiments have shown that MEnt (but not LEnt) is involved in spatial learning (Sewards and Sewards, 2003). In contrast, LEnt transmits non-spatial information to the hippocampal formation, related to the content of an experience, and is involved in non-spatial learning and memory retrieval (Knierim et al., 2013; Stouffer and Klein, 2013; Tanninen et al., 2013). While the MEnt receives input from the CA1, subiculum, presubiculum/parasubiculum (all of which also contain place or grid cells; Boccara et al., 2010), and from visual neocortical areas related to the dorsal visual stream (the “where” pathway) involved in processing spatial visual information on object location (Wang et al., 2011), the LEnt receives input from visual areas of the temporo-occipital neocortex and/or perirhinal cortex (including area 35) related to the ventral visual stream (the “what” pathway), involved in object identification and recognition (Sewards and Sewards, 2003; Canto et al., 2008; Wang et al., 2011). In both rodents and cats, both the LEnt and MEnt receive direct olfactory bulb input, although the LEnt is the preferential target (Room et al., 1984; Witter and Amaral, 2004). In rodents, LEnt shows important reciprocal connections with the pallial amygdala and possibly provides the amygdala with complex “contextual” information relevant for behavior (McDonald and Mascagni, 1997), but the involvement of MEnt in such connections is very modest (Sewards and Sewards, 2003). Both MEnt and LEnt receive weak auditory input from the temporal neocortex, and are reciprocally connected with areas of the cingulate, retrosplenial, and frontal neocortex (Sewards and Sewards, 2003; Witter and Amaral, 2004).

Are these two entorhinal cortex divisions present in birds? Current data suggest that the so-called entorhinal cortex of birds (Puelles et al., 2007; Abellán et al., 2009; present work) may be comparable to mammalian MEnt (Figure 12). This cortical area, located laterally to the APHcl/CDL, receives olfactory input (Reiner and Karten, 1985; Atoji and Wild, 2014), and has often been considered a caudal continuation of the piriform cortex (for example, Atoji and Wild, 2014). However, in both mouse and chicken, the piriform cortex shows a genetic profile different from that of this avian cortical field: the piriform cortex is characterized by strong expression of *Lmo3, Lmo4*, and *Cdh10*, very weak expression of *Lhx9*, and no expression of *Lhx2* and *Lef1*; in contrast, the avian entorhinal cortex shows moderate to strong expression of *Lmo4, Lhx9, Lhx2*, and *Lef1*, while its cortical plate is nearly free of *Lmo3* and *Cdh10* (Vyas et al., 2003; Abellán et al., 2009; present data). Also, while the piriform cortex is at the surface of the nidopallium and derives from the ventral pallium (Puelles et al., 2007), the so-called avian entorhinal cortex is adjacent to the APH and lateral horn of the lateral ventricle, and appears to derive from the medial pallium (based on position and expression of *Lef1* during early development). Based on its embryonic origin, the so-called avian entorhinal cortex may be comparable to mammalian MEnt. As noted above, MEnt also receives a minor direct input from the olfactory bulb at least in some mammals (Room et al., 1984; Witter and Amaral, 2004).

In addition, the avian APHcl/CDL may also be comparable to mammalian MEnt (Figure 12). In pigeon, the APHcl/CDL is reciprocally and extensively connected with the various areas of the hippocampal formation, i.e., DG/CA3 area, APHm, APHi, and APHl (V-shaped area, DM and DL in Atoji and Wild, 2005). Moreover, large lesions involving the CDL produce visuospatial deficits suggesting a similar role to that of mammalian MEnt, although this needs confirmation by smaller lesions or specific inactivation of APHcl/CDL (discussion in Atoji and Wild, 2005). The observed deficits are consistent with the inputs to APHcl/CDL from the visual hyperpallium (Figure 5 of Atoji and Wild, 2005), which has been involved in the “where” analysis of the information (Watanabe et al., 2011). Moreover, the lateral part of APHcl/CDL also receives direct input from the olfactory bulb (Reiner and Karten, 1985; Atoji and Wild, 2014), and for this reason it has been compared to the entorhinal cortex of mammals (Redies et al., 2001; Suárez et al., 2006). Curiously, both the avian APHcl/CDL and the mammalian MEnt include cell aggregates or patches showing neurochemical features different from the surrounding area (birds: Redies et al., 2001; Kovjanic and Redies, 2003; Suárez et al., 2006; mammals: Witter and Amaral, 2004). These patches were also evident in our chicken material at P0 as areas of the cortical plate free of *cLmo3* expression. They appear to be formed by cells having the same embryonic birth date and expressing the same types of cell adhesion-mediating cadherins (Redies et al., 2001; Kovjanic and Redies, 2003; discussed by Suárez et al., 2006). The connections and functional significance of these patches remain unknown.

On the other hand, it is uncertain whether the avian field called dorsolateral caudal pallium (DLP) is or is not comparable to the dorsolateral caudal pallial field that produces LEnt in mammals, even if they occupy similar topological positions and share some similar molecular features (for example, in general weak or moderate expression of *Lhx2, Lhx9, Lmo3*, and *Lmo4*; present data; Figure 12). The avian DLP is relatively large, and has a cortical-like area at its surface that extends ventrally (Puelles et al., 2007; called caudodorsolateral pallium or CDL by these authors), but apparently does not receive any direct olfactory input (Atoji and Wild, 2014; called temporo-occipito-mesencephalic area or TPO by these authors). This general field receives different types of information from several pallial areas, including the entopallial belt (involved in the what analysis of the information, Watanabe et al., 2011) and the mesopallium (Figure 9 of Atoji and Wild, 2005), and it projects to the avian pallial amygdala (in particular, the caudolateral nidopallium and the arcopallium) and the basal ganglia (Veenman et al., 1995; Kröner and Güntürkün, 1999). It is also connected reciprocally with the hippocampal formation, but less so than the APHcl/CDL (Atoji and Wild, 2005). It would be interesting to investigate whether there is a structure comparable to avian DLP in reptiles, which would contribute to understand its homology across amniotes. For this purpose, it is necessary to have a molecular marker (or a clear combination of them) specific of this pallial sector.

### Conflict of interest statement

The authors declare that the research was conducted in the absence of any commercial or financial relationships that could be construed as a potential conflict of interest.

## Supplementary material

We provide two supplementary figures with additional details in the expression of Lef1 and Prox1 in the mouse and chicken forebrain.

## Abbreviations

A: arcopallium (part of pallial amygdala; VLP derivative) (chicken)
ac: anterior commissure
Amyg: amygdala
AHi: amygdalo-hippocampal transition area
AHitr: amygdalo-hippocampal transition area (chicken)
APH: parahippocampal area (chicken)
APHcl: caudolateral APH (same as CDL)
APHi: intermediate APH
APHl: lateral APH
APHm: medial APH
APHr: rostral APH
APHre: ectopic part of APHr
APir: amygdalo-piriform transition area
BC: basal amygdalar complex (part of pallial amygdala)
CA: Ammon's horn fields (CA1, CA2, CA3)
can: CA neuroepithelium
cc: corpus callosum
CDL: corticoid dorsolateral area (same as APHcl) (chicken)
chp: choroid plexus
Cg: cingulate neocortex
cMEnt: caudal part of MEnt
CPu: caudoputamen
CR: Cajal-Retzius cells
cxh: cortical hem
DG: dentate gyrus
dgm: dentate gyrus migratory cells
dgn: DG neuroepitleium
DLP: dorsolateral caudal pallium
DLPco: dorsolateral caudal pallium, core nucleus (chicken)
DMH: dorsomedial hypothalamus
DP: dorsal pallium
EMT: prethalamic eminence
Ent: entorhinal cortex
GP: globus pallidus
H: hyperpallium (DP derivative) (chicken)
Hb: habenula
Hi2: hippocampal area 2 (dorsal part of V-shaped area; defined by Puelles et al., 2007)
ic: internal capsule
IG: *indusium griseum*
Ins: insular cortex
iz: intermediate zone (deepest part of the mantle, containing migratory cells)
LEnt: lateral entorhinal cortex
lfb: lateral forebrain bundle
LGE: lateral ganglionic eminence
LP: lateral pallium
LS: lateral septum
LSt: lateral striatum (chicken)
m: mantle
m^d^: deep part of the mantle
M: mesopallium (LP derivative) (chicken)
Me: medial amygdala
mes: mesoderm
MEnt: medial entorhinal cortex
MGE: medial ganglionic eminence
MP: medial pallium
N: nidopallium (VP derivative) (chicken)
NCL: caudolateral nidopallium
NCx: neocortex
o: outer or marginal zone
OB: olfactory bulb
Olf: olfactory areas
Pir: piriform cortex
PMCo: posteromedial cortical amygdalar area (part of pallial amygdala)
PO: preoptic area
PSe: pallial septum
RB: retrobulbar area
rp: roof plate
RS: retrosplenial neocortex
S: subiculum
Se: septum
sn: subiculum neuroepithelium
Sp: subpallium
St: striatum
svz: subventricular zone
tch: choroid tela
Te: temporal neocortex
Th: thalamus
v: ventricle
V: V-shaped area (chicken)
VLP: ventrolateral caudal pallium
VP: ventral pallium
vz: ventricular zone.
