# Supplementary material for: Combinatorial expression of Lef1, Lhx2, Lhx5, Lhx9, Lmo3, Lmo4, and Prox1 helps to identify comparable subdivisions in the developing hippocampal formation of mouse and chicken
Source: Front Neuroanat. 2014 Jul 4;8:59. doi: 10.3389/fnana.2014.00059 (PMC4082316; doi:10.3389/fnana.2014.00059)
Supplement: Supplementary file 1 [file DataSheet1.PDF]

Supplementary Material

Combinatorial expression of *Lef1*, *Lhx2*, *Lhx5*, *Lhx9*, *Lmo3*, *Lmo4*, and *Prox1* helps to identify comparable subdivisions in the developing hippocampal formation of mouse and chicken

Antonio Abellán, Ester Desfilis, Loreta Medina *

Laboratory of Brain Development and Evolution, Department of Experimental Medicine, University of Lleida, Institute of Biomedical Research of Lleida (IRBLleida), 25198 Lleida, Spain

*** Correspondence:** Loreta Medina, Ph.D., Laboratory of Brain Development and Evolution, Facultat de Medicina, Universitat de Lleida, Edifici Biomedicina I - IRBLleida, Avda. Alcalde Rovira Roure 80, Lleida 25198, Catalunya, Spain.
loreta.medina@mex.udl.cat

1. Supplementary Figures

Here we provide two figures with additional details on the *Lef1* and *Prox1* expression in the forebrain not shown in the main article.

1.1. Suplementary Figures

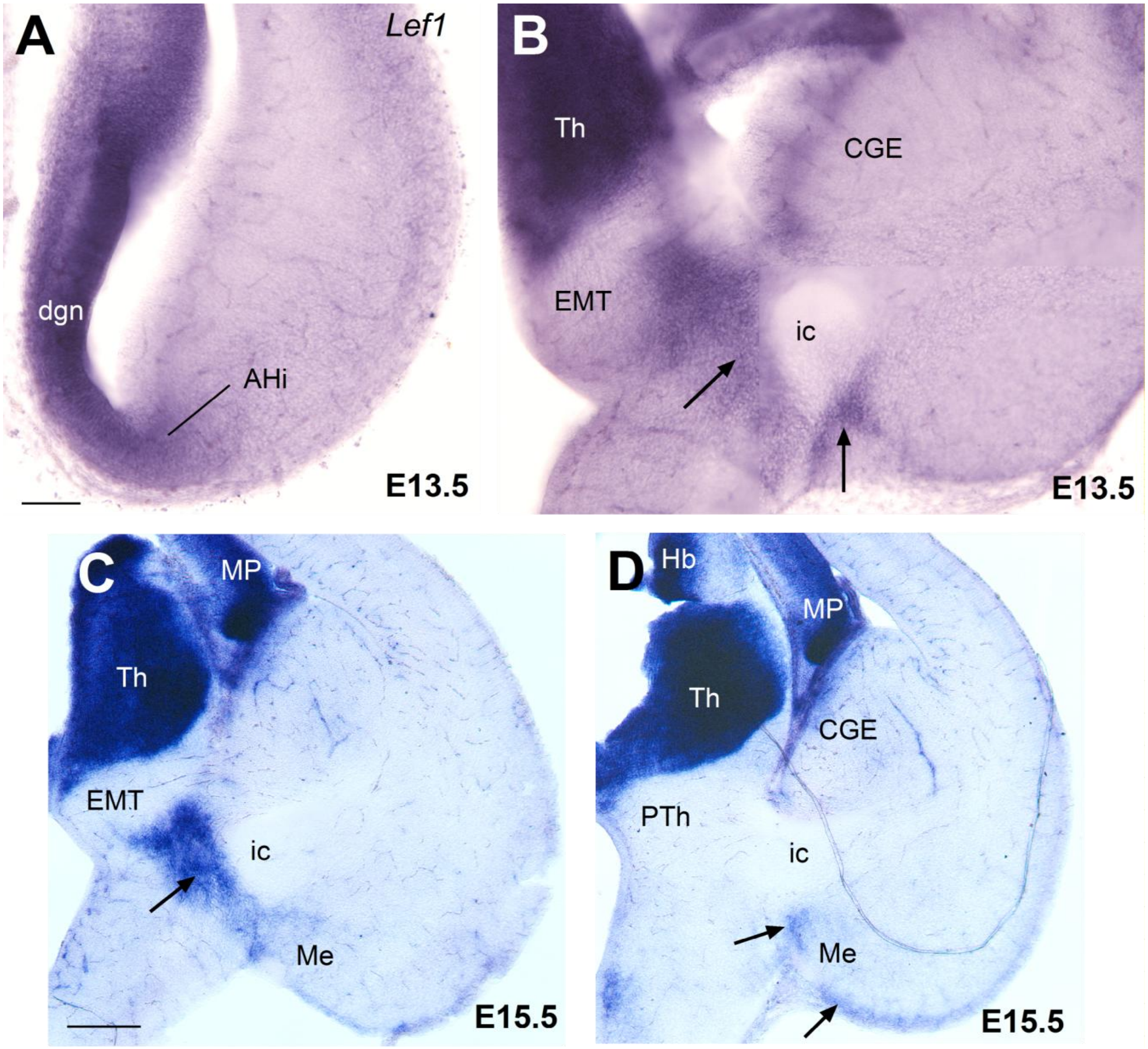

Supplementary Figure 1. Expression of *Lef1* in the mouse embryonic forebrain. Details of digital images of coronal sections of mouse embryonic telencephalon (E13.5, E15.5), showing *Lef1* expression in the amygdalo-hippocampal transition area and in a group of cells extending from the prethalamic eminence into the telencephalon (arrows). Scale bar: A = 250 microns (applies to A, B); C = 1 mm (applies to C, D). **Abbreviations:** AHi, amygdalo-hippocampal transition area; dgn, dentate gyrus neuroepithelium; EMT, prethalamic eminence; ic, internal capsule; Hb, habenula; Me, medial amygdala; MP, medial palium; PTh, prethalamus; Th, thalamus.

1.2. Suplementary Figures

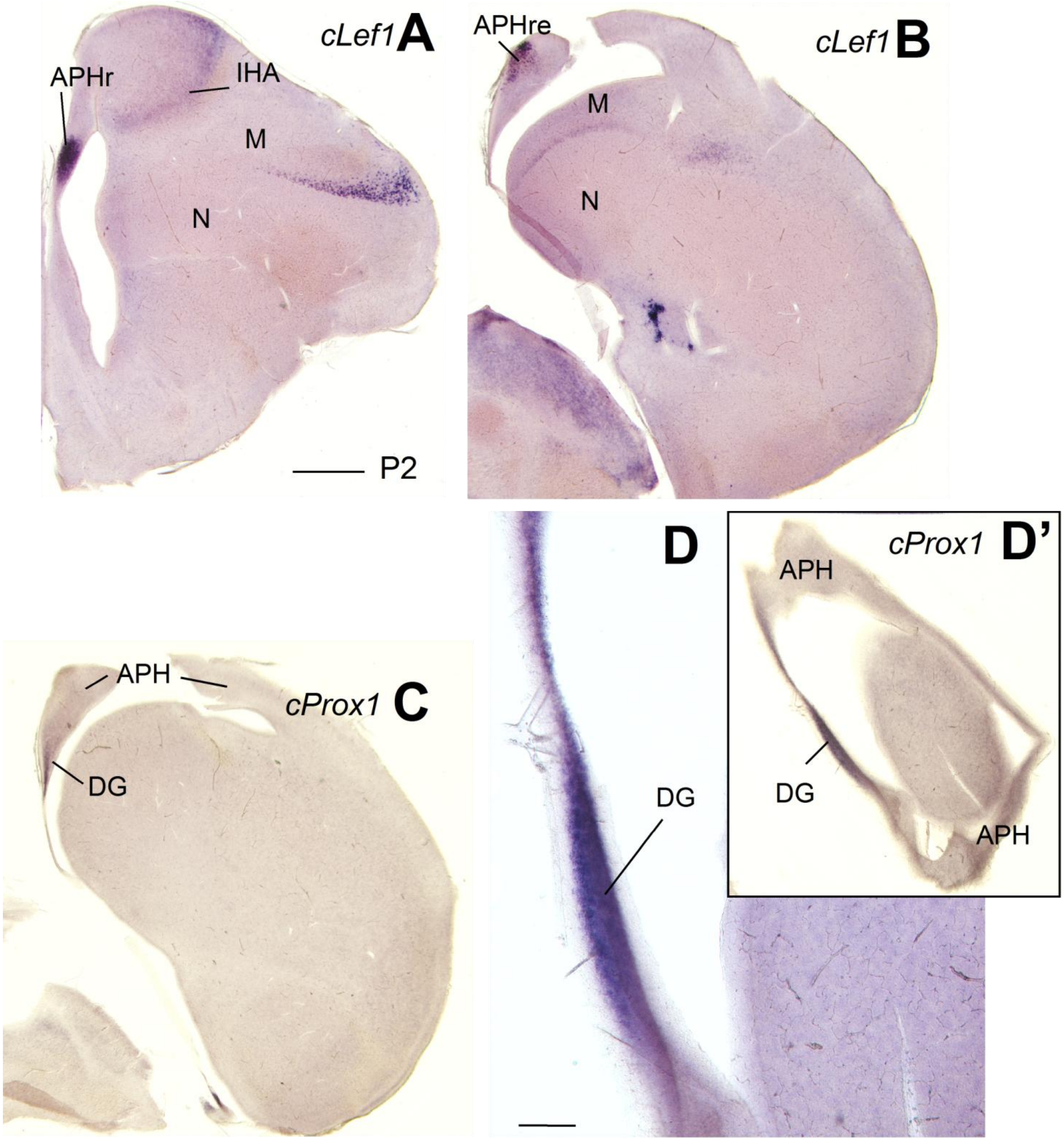

Supplementary Figure 2. Expression of *cLef1* and *cProx1* in the chicken medial pallium. Details of digital images of coronal sections of chicken telencephalon (at P2), hybridized for *cLef1* or *cProx1*. Note the expression of cLef1 in the APHr, and the expression of cProx1 in the dentate gyrus. Scale bar: A = 1 mm (applies to A-C, D'); D = 250 microns. Abbreviations: APH, parahippocampal area; APHr, rostral APH; APHre, ectopic part of APHr; DG, dentate gyrus; IHA, interstitial nucleus of the apical hyperpallium; M, mesopallium; N, nidopallium.
